# An epidemiological modeling framework to inform institutional-level response to infectious disease outbreaks: a Covid-19 case study

**DOI:** 10.1038/s41598-024-57488-y

**Published:** 2024-03-27

**Authors:** Zichen Ma, Lior Rennert

**Affiliations:** 1 Department of Mathematics, Colgate University, Hamilton, NY USA; 2 Center for Public Health Modeling and Response, Department of Public Health Sciences, Clemson University, 517 Edwards Hall, Clemson, SC 29634 USA

**Keywords:** Infectious diseases, Influenza virus, Viral infection, Health care, Public health

## Abstract

Institutions have an enhanced ability to implement tailored mitigation measures during infectious disease outbreaks. However, macro-level predictive models are inefficient for guiding institutional decision-making due to uncertainty in local-level model input parameters. We present an institutional-level modeling toolkit used to inform prediction, resource procurement and allocation, and policy implementation at Clemson University throughout the Covid-19 pandemic. Through incorporating real-time estimation of disease surveillance and epidemiological measures based on institutional data, we argue this approach helps minimize uncertainties in input parameters presented in the broader literature and increases prediction accuracy. We demonstrate this through case studies at Clemson and other university settings during the Omicron BA.1 and BA.4/BA.5 variant surges. The input parameters of our toolkit are easily adaptable to other institutional settings during future health emergencies. This methodological approach has potential to improve public health response through increasing the capability of institutions to make data-informed decisions that better prioritize the health and safety of their communities while minimizing operational disruptions.

## Introduction

The Covid-19 pandemic has caused major devastation and disruption globally. Institutions, including industry, health systems, and educational institutions, faced the particularly difficult task of operating during Covid-19^1–4^. Many public health guidelines to mitigate Covid-19 spread were undeveloped at the time such institutions reopened (e.g., pre-arrival testing for university students)^5^. While disease mitigation policies implemented by governments in broad geographic regions were effective^6^, policies informed by state or county data were insufficient and/or inefficient for disease mitigation at the local level^7,8^. Population characteristics in institutes of higher education (IHE) can be substantially different in terms of social networks and health seeking behavior relative to the general population^9^. For example, standard mitigation policies, including social distancing and masking, were not effective for preventing outbreaks in university student populations due to high social contacts and congregated housing^10^.

Institutions with flexibility and ability to implement mitigation measures tailored to their populations have utilized predictive modeling at the local level to guide decision making throughout the pandemic. IHE implemented predictive models to inform testing strategies, mask and vaccine mandates, online instruction, and other mitigation strategies to help curb disease transmission in their student and employee populations^11–14^. Accurate models are especially useful for IHE in the United States (US) and abroad, since (1) IHE students, faculty, and staff account for 7% of the US population and indirectly impact tens of millions including families and local communites^13^, (2) increased disease transmission among students due to increased social contacts and congregated living^10^, and (3) IHE are able to implement mitigation policies and behavioral interventions^13^.

Several predictive Covid-19 models have been developed since the onset of the pandemic for case projections and intervention evaluation in other institutional settings^15^, including healthcare facilities^16^, long-term care facilities^17^, and K-12 schools^18–20^. However, many of these models rely on input parameters derived from broad geographic regions which can lead to inaccurate projections for local populations^7^. When models are not tailored to local populations, uncertainty in local-level input parameters, including initial model states (e.g., population immunity)^21^, disease transmission (e.g., vaccine protection)^9^, human behavior (e.g., voluntary testing compliance)^22^, and the unpredictable nature of the pandemic^23^, further amplify model inaccuracy^24^. While predictive models can be useful for comparing the relative effectiveness of interventions^13,25,26^, inaccurate point estimates for disease incidence can ultimately complicate institutional decision making and policy^27^. Accurate case projections are needed to inform institutional resource planning and procurement, such as testing kits, isolation beds, ventilators, staffing, etc.^5,11,28^. Fortunately, many large institutions have rich data sources that can directly estimate input parameters to guide predictive models. Such modeling frameworks allow institutions to make informed decisions that better prioritize the health and safety of their local communities while minimizing operational disruptions.

In this study, we describe the development and implementation of a novel epidemiological modeling toolkit for institutional Covid-19 surveillance, prediction, resource procurement, and evaluation of institutional mitigation strategies. This modeling framework formed the basis for Clemson University’s decision-making throughout the Covid-19 pandemic. A novel feature of our toolkit is the utilization of the entire pipeline of institutional data in all stages of the modeling framework, including (1) estimation of local disease surveillance metrics, (2) statistical modeling of local disease transmission dynamics, and (3) compartment-based modeling framework for Covid-19 prediction based on input parameters estimated in (1), (2), and publicly available data. We argue that this strategy helps minimize uncertainties in model input parameters presented in the broader literature, and demonstrate that this institutional-level modeling toolkit can accurately predict the number of Covid-19 cases, inform resource procurement, and evaluate the relative effectiveness of mitigation measures. Moreover, the generalized version of this (publicly available) toolkit can yield reasonably accurate predictions in other university settings. The input parameters of this toolkit are adaptable to other institutional settings during (respiratory) infectious disease outbreaks.

## Results

### Model structure

**Figure 1:**
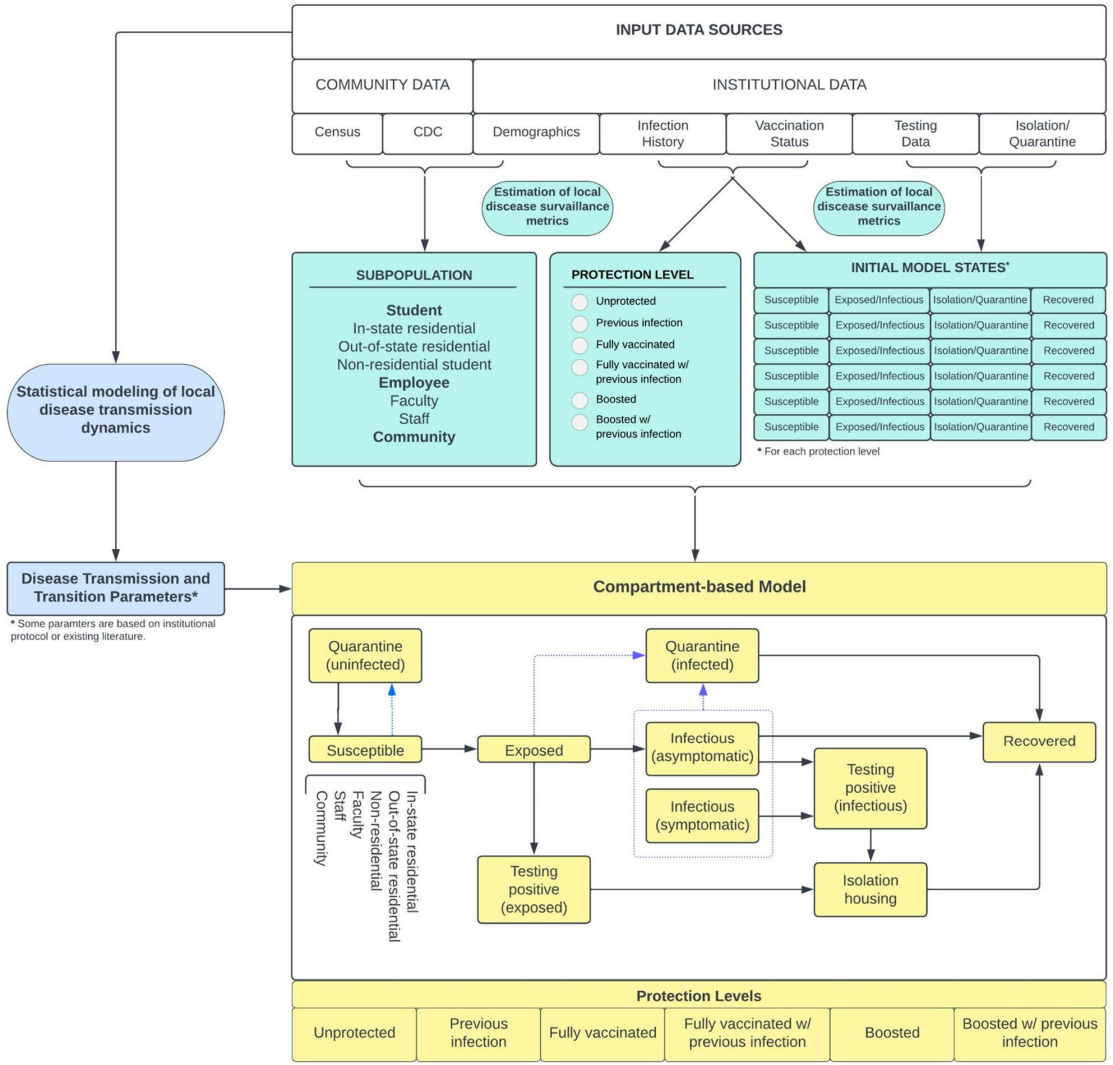
Modeling framework. The modeling framework of the toolkit includes estimating local disease surveillance metrics, statistical modeling of local disease transmission dynamics, and compartment-based modeling framework for Covid-19 prediction based on estimated input parameters and publicly available data.

For each affiliate subpopulation (in-state residential student, out-of-state residential student, non-residential student, faculty, staff, community), individuals were assigned into an immunity (or protection) level: no immunity, previous SARS-CoV-2 infection only, full vaccination, boosted, full vaccination with previous infection, boosted with previous infection (additional detail provided in Methods and Supplementary Text). Within each affiliate/immunity level subpopulation, individuals were placed in one of the compartments detailed in Fig. 1. Details on statistical models, estimation of protection parameters, disease transmission and transition parameters, including those derived from scientific literature or institutional protocol, is provided in Methods Supplementary Text. Initial compartment states and disease transmission/transition parameters were then inserted as input parameters into the compartment-based modeling (CBM) framework. The CBM provides predictions of the weekly number of cases and infection rates, the daily number of isolated individuals, and the daily number of isolated and quarantined individuals (by affiliate subpopulation). In addition, the toolkit also displays a summary of the initial states and the estimated disease transmission dynamics. A step-by-step tutorial of this publicly available toolkit is included as a supplement to this article.

### Main analysis—Clemson University Analysis (Spring 2022)

There were 27,516 individuals in the main-campus population, including 22,634 students (4853 in-state residential students, 2265 out-of-state residential students, 15,516 non-residential students) and 4882 employees (1611 faculty, 3271 staff). Also included were 17,681 from the local community^29^. The residential population was split into in-state and out-of-state, since out-of-state residential students were more likely to use university-provided housing (if SARS-CoV-2 positive) due to travel restrictions. Students and employees were subject to mandatory arrival testing and weekly surveillance testing during in-person instruction. Initial values for students and employees in each compartment are based on empirical data with adjustments for underreporting (Table S1) at the start of the prediction period (January 10, 2022). During this period, the omicron BA.1 variant accounted for 99.2% of SARS-CoV-2 cases in South Carolina^9^.

**Figure 2:**
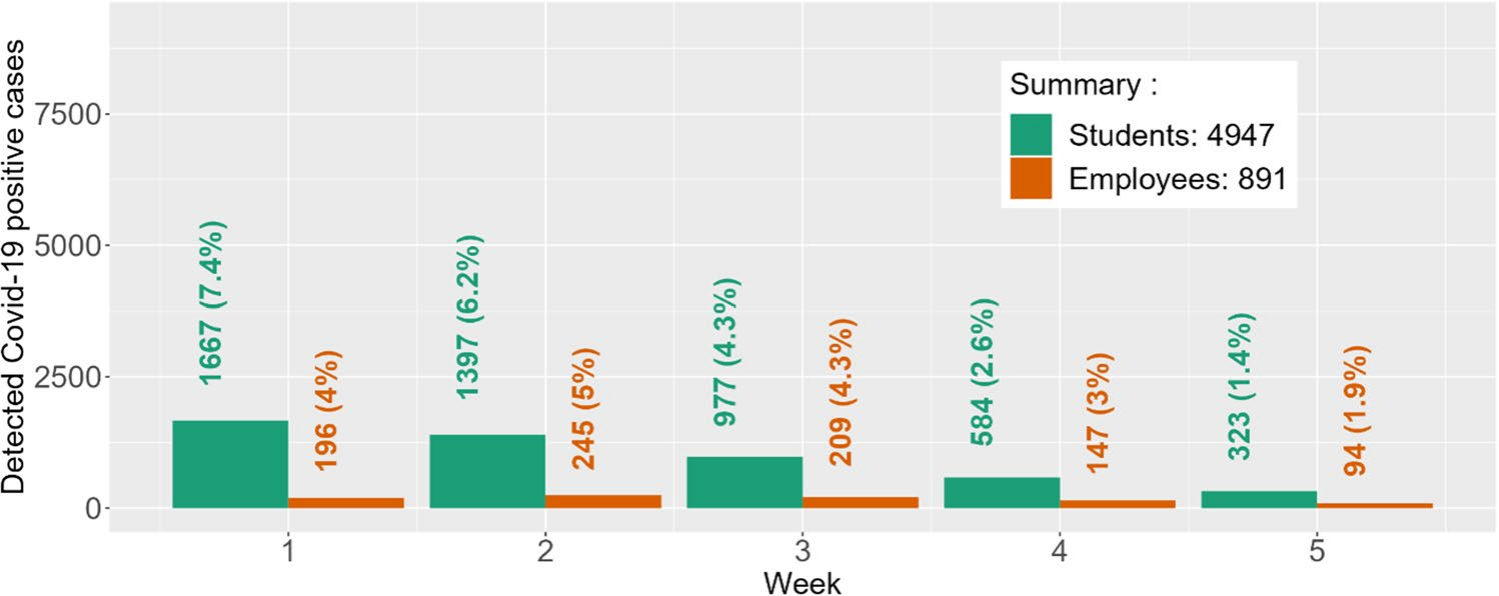
Predicted student and employee Covid-19 cases (percent of population) under weekly testing during first 5 weeks of Spring ’22 at Clemson University. Week 1 started on January 10, 2022. Over the five weeks, the observed student cases were 2035, 1678, 732, 296, and 135, respectively (total observed cases = 4876; % agreement = 98.6%). The observed employee cases over the five weeks were 308, 264, 160, 90, and 54, respectively (total observed cases = 876; % agreement = 93.2%). The % Agreement is calculated as *min(O*_*ij*_*,P*_*ij*_*)*/*max(O*_*ij*_*,P*_*ij*_*)*, where *O*_*ij*_ and *P*_*ij*_ are the observed and predicted Covid-19 cases in week *i* for subpopulation *j*.

Estimated student and employee disease prevalence at baseline (January 6th through 9th) was 15.1% and 4.8%, respectively. The number of individuals in each immunity level, along with estimated protection by immunity level, is provided in Table S9. The disease reproductive number for each subpopulation was validated using empirical data from the Spring and Fall 2021 semesters and published literature (Methods and Supplementary Appendix 1). Predicted SARS-CoV-2 cases under weekly surveillance testing for students and employees during the 5-week follow-up period (January 10–February 13, 2022) are provided in Fig. 2. Observed cases represent the total number of tests with positive results during the indicated prediction period. Predicted cases represent the total number of students and employees tested positive during the indicated prediction period. Total predicted student and employee cases (%) during this 5-week period was 4947 (21.9%) and 891 (19.2%). Total observed cases (%) for these populations were 4876 (21.5%) and 876 (17.9%), respectively.

Further, the percent-agreement for total detected cases was 98.6% for students and 93.2% for employees. In addition, the percent agreement for the peak number of weekly detected cases is 81.9% for students (observed N = 2035; predicted N = 1667) and 79.5% for employees (observed N = 308; predicted N = 245). The predicted peak for students concurred with the observed peak at Week 1 (Jan. 10–16), but the predicted peak for employees occurred a week later than the observed peak.

**Figure 3:**
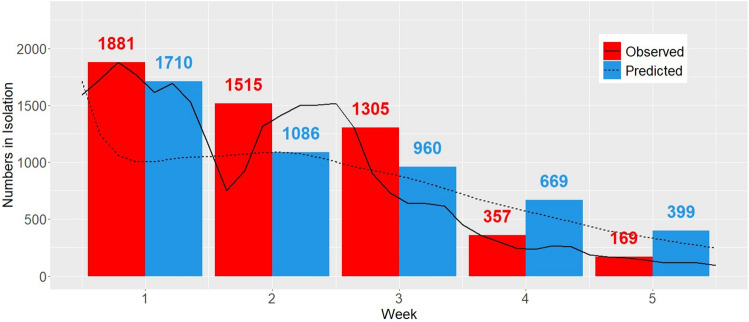
Observed and predicted number of maximum student isolation beds needed each week under weekly testing during first 5 weeks of Spring ’22 at Clemson University.

Observed and predicted students in isolation over the 5-week prediction period are presented in Fig. 3. Clemson University’s Isolation and Quarantine (I/Q) policies were based on the latest CDC guidelines^30^. We were interested in the maximum number of students in isolation, since this is directly linked to procurement of rooms. Predicted and observed peak isolation counts were 1710 and 1881, respectively, corresponding to an agreement of 91.8%. The residential population is of particular interest since this population lives in congregated housing and, therefore, cannot isolate/quarantine in place. Among residential students, predicted and observed peak isolation counts were 673 and 649 (% agreement: 96.3%). In addition, among out-of-state residential students, predicted and observed peak isolation capacity were 264 and 194 (% agreement: 73.5%).

There was some daily variation in observed peak isolation (relative to predicted). Of note is the discrepancy between peak capacity towards the end of week 2 (predicted peak: 1086, observed peak: 1515; agreement: 72%). This was primarily due to daily fluctuation in student testing schedules and limited weekend testing, which was not incorporated into the modeling framework.

Prior to the start of each semester, we were tasked with evaluating the impact of testing strategies on mitigating disease spread. This has been extensively studied for previous variants (prior to omicron), which have concluded that testing at least once per week is sufficient for mitigating disease spread^12,13^. Here we compared the projected cases during the five-week projection period under four different testing strategies: weekly, bi-weekly, monthly, and voluntary testing. We consider two time periods: Spring 2022 semester (omicron BA.1 variant) and Fall 2022 semester (omicron BA.5 variant).

**Figure 4:**
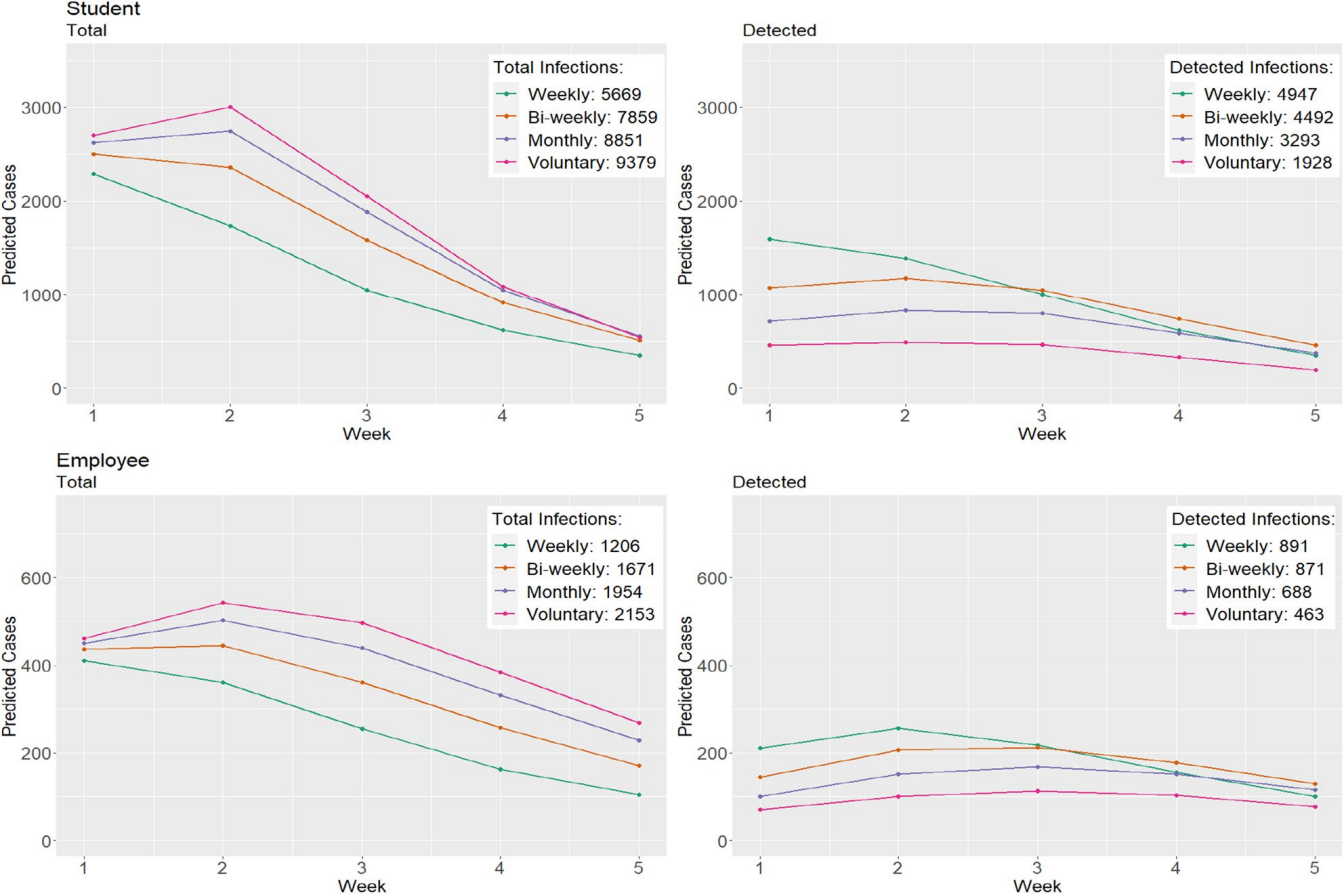
Comparison of predicted cases under different SARS-CoV-2 testing strategies at Clemson University during first 5 weeks of Spring ’22.

For voluntary testing, we estimated that only 10% of total SARS-CoV-2 infections would be detected for students and 15% for employees. Results for the Spring 2022 semester are presented in Fig. 4. Weekly testing led to 1.10, 1.50, and 2.57 times more detected student cases compared to bi-weekly, monthly, and voluntary testing (weekly: 4947, bi-weekly: 4492, monthly: 3293, voluntary: 1928) and 1.02, 1.30, and 1.92 times more detected employee cases compared to bi-weekly, monthly, and voluntary testing (weekly: 891, bi-weekly: 871, monthly: 688, voluntary: 463), respectively. The opposite was true for total cases (both symptomatic and asymptomatic). Here, voluntary testing led to 1.65, 1.19, and 1.06 times more total student cases compared to weekly, bi-weekly, and monthly testing (weekly: 5669, bi-weekly: 7859, monthly: 8851, voluntary: 9379) and 1.79, 1.29, and 1.10 times more total employee cases compared to weekly, bi-weekly, and monthly testing (weekly: 1206, bi-weekly: 1671, monthly: 1954, voluntary: 2153), respectively. Based on these findings, Clemson University continued with weekly testing during the first half of the Spring 2022 semester. While similar (relative) trends were observed when comparing testing strategies prior to the Fall 2022 semester (Fig. S1), overall predicted cases were lower under the four testing strategies. This is primarily due to the substantial increase in population immunity from the Omicron BA.1 variant, which resulted in a lower susceptible population^9,31^.

### Extension to other institutions and time periods

We generalized the modeling framework above to obtain predictions in three other settings. The first two projections were conducted for the University of Georgia (UGA) and Pennsylvania State University (PSU) during the Spring ’22 semester. These institutions were natural choices for external validation, as both are land-grant universities with publicly accessible data on weekly Covid-19 cases. Because institutional vaccination data was unavailable, we used literature-based estimates of vaccine protection for these populations (Table S7). The third set of projections utilized the generalized modeling framework for predictions at Clemson University during the Fall 2022 semester (omicron BA.5 variant).

For UGA and PSU, we obtained the total number of students and employees in each university and the number of infections during the week prior to the prediction start (January 10th, 2022) from institutional websites and Covid-19 dashboards^32,33^. Because UGA and PSU did not implement mandatory surveillance testing, reported Covid-19 cases are from voluntary testing and therefore overall case prevalence is underreported. We adjust these estimates by an (estimated) constant to obtain the asymptomatic/undetected infection rate at baseline (see Methods and Supplementary Appendix 1). Due to lack of information on vaccination and previous infection rates, we estimate these quantities using a combination of Clemson institutional data and data from the Centers for Disease Control and Prevention (CDC)^34^. The calculation of subpopulation sizes and other details are provided in Supplementary Appendix 1.

We used our toolkit to predict the number of weekly cases and the maximum number of weekly cases for university students and employees at UGA and PSU over the 5-week period (January 10 to February 13, 2022). The results are provided in Table 1. Additional information on the initial values, estimated individuals in each protection level, and model input parameters is given in the Supplementary Materials (Table S3–4, S7, S10–11). The percent agreement for the total detected cases over the prediction period was 96.7% for UGA (observed N = 2550; predicted N = 2467) and 89.5% for PSU (observed N = 1708; predicted N = 1983). In addition, we examined the peak number of cases during the five weeks, as this informs decisions on health resources (isolation beds, meals, medical staff, contact tracers, etc.). The percent agreement for peak weekly cases was 65.4% (observed N = 1003; predicted N = 656) for UGA and 75.6% (observed N = 631; predicted N = 477) for PSU. In both scenarios, the predicted peak occurred one week after the observed peak.

**Table 1:** Comparison of observed and predicted cases (detected) at UGA and PSU during first 5 weeks of Spring ’22. The % Agreement is calculated as *min(O*_*i*_*,P*_*i*_*)*/*max(O*_*i*_*,P*_*i*_*)*, where *O*_*i*_ and *P*_*i*_ are the observed and predicted Covid-19 cases in week *i*.

	Observed cases	Predicted cases	% Agreement
University of Georgia			
1/10–1/16	**1003**	529	
1/17–1/23	929	**656**	
1/24–1/30	363	526	
1/31–2/6	166	326	
2/7–2/13	89	329	
Total	2550	2467	96.7%
Pennsylvania State University			
1/10–1/16	539	286	
1/17–1/23	**631**	455	
1/24–1/30	340	**477**	
1/31–2/6	128	436	
2/7–2/13	70	329	
Total	1708	1983	89.5%

### Clemson University Analysis (Fall 2022)

We used the model to project the number of cases and number in isolation for the beginning of the Fall ’22 semester (August 24–September 27, 2022) at Clemson University, where the BA.5 omicron variant was the dominant SARS-CoV-2 in the population^35^. The notable difference, compared to the main analysis, is that the University implemented a voluntary/symptomatic testing strategy mid-way through the Spring ’22 semester. Consequently, many infections between this period and the Fall ’22 semester went unreported. We therefore imputed estimates of unreported infections during periods of voluntary testing (December 12, 2021–January 2, 2022 and April 2–May 22, 2022) into the previously infected compartments. Estimated unreported infections occurring in the 90-day window between May 23, 2022 and the start of follow-up were imputed into the recovered compartment. Estimated unreported infections during the 90-day window prior to start of the Fall 2022 semester (May 23–August 21, 2022) were added to the recovered compartment. Details on the estimation procedures are provided in Methods and Supplementary Text. Due to lack of mandatory pre-arrival or arrival testing which resulting in small sample sizes at the semester start, these predictions no longer utilize statistical models to estimate protection from vaccine or previous infection. Rather, the protection parameter for each protection level was set according to existing literature^36^. Full details on initial values and model input parameters for this analysis are provided in Supplementary Materials (Table S5 and S8, respectively).

**Figure 5:**
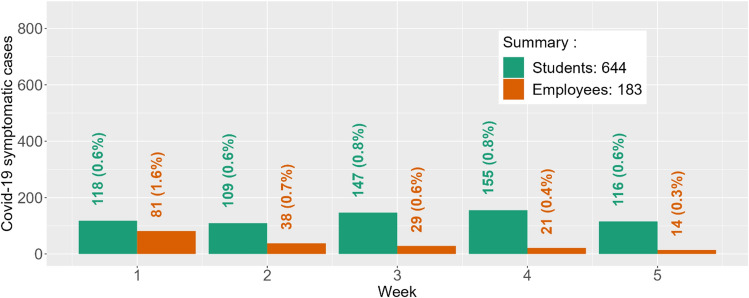
Predicted student and employee Covid-19 cases under voluntary testing during first 5 weeks of Fall ’22 at Clemson University. Week 1 started on August 24, 2022. Over the five weeks, the observed student cases were 197, 254, 115, 49, and 21, respectively (total observed cases = 636; % agreement = 98.8%). The observed employee cases over the five weeks were 32, 33, 22, 15, and 16, respectively (total observed cases = 118; % agreement = 64.5%). The % Agreement is calculated as *min(O*_*ij*_*,P*_*ij*_*)*/*max(O*_*ij*_*,P*_*ij*_*)*, where *O*_*ij*_ and *P*_*ij*_ are the observed and predicted Covid-19 cases in week *i* for subpopulation *j*.

There were 24,264 individuals in the main-campus population, including 19,082 students (4670 in-state residential students, 2323 out-of-state residential students, 12,089 non-residential students) and 5183 employees (1754 faculty, 3429 staff). Estimated student and employee disease prevalence at baseline was 29.3% and 14.1%, respectively. The number of individuals in each immunity level, along with estimated protection by immunity level, is provided in Table S12. Predicted Covid-19 symptomatic infections for students and employees during the follow-up period are provided in Fig. 5.

Predicted student and employee symptomatic infections (% of population) during this 5-week period were 644 (3.4%) and 183 (3.6%). Total observed cases (% of population) for these populations were 636 (3.3%) and 118 (2.2%), respectively. Figure 5 provides a weekly comparison between the projected and observed number of detected cases during the five-week prediction period. The percent agreement for total detected cases was 98.8% for students and 64.5% for employees. In addition, the percent agreement for the peak number of weekly detected cases is 61.0% for students (observed N = 254; predicted N = 155) and 40.7% for employees (observed N = 33; predicted N = 81). The predicted peak occurred two weeks later than the observed peak for students and one week prior to the observed peak for employees.

### Input parameter sensitivity

Sensitivity of predictions to model input parameters have been extensively studied for Covid-19^12,13,37,38^. In this section, we explore sensitivity to some of the parameters unique to our modeling framework. One novel feature is accounting for protection from previous infection. We conduct a sensitivity analysis ignoring this assumption by assuming no protection from previous infection. In all settings, cases were substantially overestimated (range: 5.7–62.7%, see Table S13–S15). At Clemson University, ignoring this assumption would have led to an estimated increase in necessary I/Q capacity of 137.7% during the Fall 2022 semester, but is estimated to have had no impact on I/Q during the Spring 2022 (which is expected, since previous infection offered little protection against the omicron BA.1 variant).

In addition, there are many individuals whose infection history was unknown. We overcome this limitation by estimating the number of individuals who were previously infected by omicron but not recorded in institutional databases. If we ignore this assumption and assume that no previously infected individuals were missed, this lead to substantial overestimation in the number of predicted cases (range across scenarios: 64.2% to 343.0%, see Table S13–S15). At Clemson University, ignoring this assumption would have led to an estimated increase in necessary I/Q capacity of 39.8% (Spring 2022) and 96.5% (Fall 2022).

The proportion of individuals who voluntarily seek a Covid-19 test when infected is an important assumption in prediction modeling. Increasing the proportion of infectious individuals who seek a Covid-19 test from our assumption of 10% to 20% for students and from 15 to 30% for employees, the predicted number of cases in Spring 2022 when mandatory weekly testing was implemented, increased by 0.3% for students and 2.2% for employees. This result is expected, as increasing voluntary testing rates under mandatory weekly testing would only impact how soon symptomatic individuals would seek a test after infection, but would not impact their decision to obtain a test. In Fall 2022, when mandatory testing was no longer in place, doubling the proportion of infectious individuals who seek a Covid-19 test would have led to an estimated 77.0% increase in detected cases among students and a 69.4% increase among employees.

At multiple periods throughout the pandemic, this toolkit was used to inform the removal of mitigation measures, including social distancing requirements, mask mandates, and mandatory testing. Because it is difficult to model the precise impact of a masking or social distancing mandate, we instead compared predicted cases under two scenarios: strong effect of the mitigation measure versus no effect of the mitigation measure. For example, our team was tasked with evaluating the impact of the classroom mask mandate mid-way through the Spring 2022 semester (after the omicron BA.1 wave had resided). To evaluate sensitivity of model predictions to changes in mitigation measures, we incorporated six daily time steps (4 h each) into our model. Under the reference setting (corresponding to 4 weekday time steps), which was assumed to represent non-work or school hours, we assumed minimal contact between students and employees or community members^13^. During class hours (1 weekday time step) and work/study hours (1 weekday time step), we assumed increased contact between students and faculty, but decreased rates of transmission. Weekend time steps assumed increased transmission rates and higher contact rates between students and employees with community members. Transmission rates across time steps were calibrated to correspond to reference transmission levels (on average). Full details on the contact network matrix and transmission rates by time step are provided in Supplementary Appendix 1.

Assuming masks decrease disease transmission by 50%^39^, we conservatively assumed absence of a mask mandate would double transmission during the classroom time step. During the first 5 weeks of the Spring 2022 semester, removing the mask mandate would have led to an estimated increase of 171 student cases and 119 employee cases. During the first 5 weeks of the Fall 2022 semester, implementing the mask mandate would have led to a decrease of 15 student cases and 9 employee cases. Negligible differences in Fall 2022 are not surprising given that most high-density social interactions occur outside of the classroom. Since Covid-19 prevalence was relatively low compared to previous states of the pandemic and a high majority of the population had protection from previous infection or vaccination, a mask mandate implemented during a period of the day in which social contact was reduced would have minimal impact on overall disease spread.

Our results were not overly sensitive to the choice of contact network structure. To assess sensitivity to assumptions of contact network, we increased contact rates between students and employees/community members by 25%. This led to a decrease of 21 student cases and an increase of 13 employee cases in Spring 2022 and a decrease of 6 student cases and an increase of 3 employee cases in Fall 2022.

## Discussion

The methodological approach applied in this study is novel in that it utilizes the entire pipeline of institutional data in all stages of the modeling framework, incorporating real-time estimation of disease surveillance and epidemiological measures based on institutional data. This institutional-level modeling toolkit can accurately predict the number of Covid-19 cases, inform resource procurement, and evaluate the relative effectiveness of mitigation measures. Therefore, through incorporation of (1) estimation of local disease surveillance metrics, (2) statistical modeling of local disease transmission dynamics, and (3) compartment-based modeling framework for Covid-19 prediction based on input parameters estimated in (1), (2), and publicly available data into the modeling framework, there models can minimize uncertainties in model input parameters presented in the broader literature. Moreover, the generalized version of this (publicly available) toolkit can yield reasonably accurate predictions in other university settings. The input parameters of this toolkit are easily adaptable to other institutional settings during (respiratory) infectious disease outbreaks.

The modeling framework presented in this study was directly used to inform resource allocation and decision making around both implementing, and removing, mitigation measures at Clemson University beginning in the Fall 2020 semester. Early versions of this modeling framework helped inform the number of Covid-19 testing kits needed for arrival and surveillance testing strategies, phased reopening strategies, and the number of necessary isolation/quarantine rooms prior to reopening in the fall of 2020^5,11,12^. Due to the changing nature of the pandemic, including added protection from previous infection^40^, vaccination^41^, and the introduction of new SARS-CoV-2 variants which altered disease transmission dynamics^9,42^, our toolkit was continuously modified to evaluate effective testing strategies in future semesters.

Beginning in summer of 2021, this toolkit was also used to scale back testing strategies and other mitigation measures that were projected to have a small impact on disease spread. For example, the weekly Covid-19 testing mandate for student and employee populations was not predicted to have a substantial impact on disease spread during summer 2021 due to strong protection from vaccination and previous infection combined with low disease prevalence. Findings of reduced impact of mitigation measures during periods of low disease prevalence in IHE settings are consistent with other settings^43^. The testing mandate was subsequently removed during this time, but reimplemented at the start of the Fall 2021 semester as the Delta variant began circulating^41^. The weekly testing mandate was again removed after the omicron (BA.1) wave had subsided in mid-spring of 2022.

Utilizing a contact matrix that broke down social contact patterns and disease transmission by time of day, day of week, and between student, employee, and community populations, we were able to evaluate sensitivity to additional mitigation measures including on-campus social distancing and mask mandates. For example, we projected that social distancing policies had little impact on overall transmission rates due to the majority of social interactions, and hence disease transmission, occurring off campus or in residential halls. Similarly, the toolkit showed that when disease prevalence is low and protection in the population is high, classroom mask mandates no longer had a substantial impact on overall cases due to low adherence to masking off-campus (where the majority of transmission occurs).

Utilizing the entire pipeline of Clemson Institutional Data, our toolkit was able to predict cases with high accuracy (students: 98.6%, employees: 93.2%). Furthermore, incorporating input parameter estimates based on Clemson data yielded high prediction accuracy for total Covid-19 cases at other institutions (UGA: 96.7%, PSU: 89.5%). Lower prediction accuracy for PSU relative to UGA may be explained by the relatively closer demographic similarities between Georgia and South Carolina. When replacing institutional-level estimates of disease transmission parameters with literature-based estimates, the modeling toolkit still yielded fairly high predictions for the omicron BA.5 variant during the Fall 2022 semester at Clemson University for students (accuracy: 98.8%) but overestimated total employee cases (accuracy: 64.5%).

Similar to other studies conducted prior to introduction of the Omicron variant, we found that high-frequency testing was effective in reducing SARS-CoV-2 transmission^12,13^. This finding was consistent throughout each semester despite the introduction of more transmissible variants and the introduction of effective vaccinations^41^, as the impact of higher transmission was offset by increased protection in the population^40,41^. However, the introduction of the omicron variant that plagued the nation in early 2022 complicated selection of optimal testing strategies, since increased disease transmission and lower vaccine protection^9^ reduced the effectiveness of weekly testing strategies relative to previous variants. While institutions could theoretically increase the frequency of testing, this would have required procuring additional testing kits, lab equipment, and personnel in a relatively short time period. Without sufficiently scaling up in a timely manner, which was unrealistic for many institutions in the month between introduction of the Omicron variant and the start of Spring 2022 semester, an increase in frequency of testing would have caused a significant lag in test diagnostics, thus allowing infectious individuals to transmit the disease for a longer period of time and potentially reducing the effectiveness of the testing strategy^44^.

In addition to predicting the total number of cases, the toolkit was reasonably accurate in predicting the maximum number of isolations at Clemson University during the Spring 2022 semester (90.9% accuracy) and Fall 2022 semester (79.5% accuracy). At Clemson University, this had important implications for procuring sufficient isolation/quarantine rooms between Fall of 2020 through Spring of 2022. Based on these predictions, the university procured an off-campus hotel that could house over 800 students.

Due to unavailability of isolation/quarantine data at other institutions, we predicted the peak number of weekly cases and the timing of the peak as a surrogate for total isolations each week. Prediction accuracy ranged from 79.9 to 83.3%. While reasonable for model-based predictions, the model underestimated the maximum number of weekly infections by 17–20%. Furthermore, the predicted timing of the peak was off by one week. However, this has little implications for decision making as isolation/quarantine rooms must be procured well in advance.

One of the biggest factors leading to more precise predictions was the ability of the modeling toolkit to accurately estimate initial model states and protection from previous infection. In particular, there are a substantial number of individuals in this population with unrecorded previous infections, which has a substantial impact on predictions in IHE^13^ and other settings^45^. Specifically, we showed that ignoring these features leads to underestimating the amount of immunity in the population and thus substantially overestimating the number of infections.

### Extension to other institutional settings

With some modifications, our modeling framework can be applied to other institutional settings. Large health care systems or hospitals are the most natural setting for extension, since such institutions are both impacted by, and required to respond to, health emergencies^16^. Furthermore, such institutions have agency to implement their own policies and presumably have access to most, if not all, of the necessary data sources. However, additional compartments may need to be added if the focus is on severe health outcomes (e.g., hospitalizations or deaths).

Even without the entire pipeline of institutional data, our modeling framework was fairly accurate for external predictions in IHE settings through extrapolation of Clemson institutional data or through use of publicly available CDC/Census data in conjunction with literature-based estimates for input parameters. The framework for our modeling toolkit can serve large workforces and other private or public institutions, including K-12 schools, requiring updates to initial state input parameters that reflect subpopulations in each institution. However, disease transmission and transition parameters during Omicron are unlikely reflective of current or future variants. The predictive performance of such models for new scenarios will therefore depend on the accuracy of the input parameters provided by the user. Furthermore, for each institutional setting, the current IHE-based contact network matrix would need to be updated to reflect reasonable assumptions for that institution. Additionally, as noted by a Reviewer, the risk of infection can vary by subpopulation type which would require furthermore modification of the contact matrix^46,47^. Future adaptations of this framework may benefit from leveraging digital traces and other contextual information to estimate contact networks and transmission^48–50^. In the absence of this information, one could use an “equal coupling” contact matrix^51^. However, incorrect specification may result in biased predictions.

### Extension to other diseases

Our proposed toolkit is readily adaptable to other respiratory infectious diseases. This would require data sources relevant to the disease of interest or literature-based estimates. For example, new SARS-CoV-2 variants or other respiratory viruses would require updating the disease reproductive number/transmissibility, infectivity period, level of protection in the population, and other disease transition and transmission parameters that are disease-specific. However, estimation procedures for initial model states and disease transmission parameters, along with the compartments in the prediction framework, would remain the same. For non-respiratory infectious diseases, additional modifications to the compartments would also be needed.

### Limitations

Our proposed modeling framework faces many of the limitations shared by other modeling studies. First, the high prediction accuracy of our toolkit does not imply that estimates of model input parameters and disease transmission parameters are necessarily accurate. Due to the large number of parameters, there are likely several reasonable combinations of parameters that yield similar predictions. This can have important implications to model predictions given strong sensitivity to input parameters^13^. In our framework, we attempted to minimize the impact of parameter uncertainty through estimation of influential model parameters using over 1 million data records, internal validation, and external validation through comparison to estimates in the published literature. As an extension to this modeling framework, a stochastic component can be incorporated to provide credible intervals for predicted point estimates in order to account for uncertainty in model input parameters (e.g., disease reproductive number)^13^.

Additional limitations of our modeling framework include the simplifying assumptions often made in compartment-based modeling, including homogeneity of input parameters within each subpopulation, uniform transmission rates over infectivity period that do not vary by days since infection or severity of infection, and assuming the community is a homogeneous population. To reduce the impact of homogeneous populations, we split the populations into subpopulations including non-residential and residential students (both in-state and out-of-state), faculty, staff, and community. The contact network structure for these subpopulations was based on reasonable approximations from existing literature and input from university students, faculty, staff, and administrators. However, validation of the proposed network structure is not feasible due to parameter identifiability issues previously discussed. While model predictions were not overly sensitive to the choice of contact network structure in the IHE setting of this study, such features may not translate to other institutional settings.

Due to underreporting of booster doses at Clemson University, use of Clemson vaccination data to define protection levels yields (1) a boosted group containing only a fraction of the individuals receiving a booster dose and (2) a fully vaccinated group containing a mix of fully vaccinated and boosted individuals. We therefore supplemented analyses based on Clemson vaccination data with CDC-based estimated, which yielded similar results. Given the population-averaged nature of compartment-based models, this finding is not surprising given the use of institutional data to estimate both vaccine protection and vaccination groups. Vaccine protection is estimated from this mixed population and, therefore, represents a weighted estimate of vaccine effectiveness in fully vaccinated and boosted individuals, limiting the downstream impact of misclassification on predictions.

However, prediction accuracy may not translate to future waves of the Covid-19 pandemic. For example, estimation of population-level immunity from previous infection will become more difficult given the decreasing in testing or use of at-home testing kits^52,53^. One potential solution in the absence of reliable data or estimation is to simplify the model through merging of compartments^24^. For example, merging asymptomatic and symptomatic infections into single infectious compartment, merging vaccination groups, or merging previously infected individuals into the reference compartment. While such a shift does not directly mimic the natural course of disease progression, reasonable predictions can still be obtained given that compartment-based models are population-averaged models to begin with. Studies suggest that in the absence of reliable data for model input parameters (including initial states and disease transmission/transition parameters), this strategy will result in improved prediction accuracy^24,54^. Even if prediction accuracy is reduced, previous studies have shown that evaluation of mitigation measures can be robust to variation of model input parameters^12,13^.

## Conclusions

The institutional modeling framework developed in this study is informative for disease monitoring and projections, procurement and allocation of resources, and intervention implementation, and the publicly available modeling toolkit can be directly used to guide institutional-level decision-making. Covid-19 will unlikely be the last pandemic in our lifetime. It is very possible that high impact pathogens, including coronaviruses and influenza A viruses, will emerge and reemerge^55^. The methodological approach presented here advances the field of public health preparedness and response by improving the ability of institutions to make data-informed decisions that better prioritize the health and safety of their communities while minimizing operational disruptions. Institutions must therefore be prepared and ensure that proper data collection and processing protocols are in place. In the event of a future respiratory infectious disease outbreak, our proposed modeling framework can easily be adapted to inform decision-making in large institutional settings.

## Methods

### Data collection

#### Testing

Prior to the start of each semester at Clemson University (through Spring 2022), all students and employees were required to submit a pre-arrival testing result through the COVID-19 Test Upload Tool within 10 days of in-person instruction. Accepted tests for pre-arrival testing included nasal, throat or saliva-based polymerase chain reaction (PCR) tests or antigen tests. Testing was available on campus through the University’s clinical diagnostics lab, Student Health Services, or could upload their PCR test result through an online portal. Additional details on testing protocols for the Spring ’22, Fall ’21, Spring ’21, and Fall ’20 semesters are provided elsewhere^9,12,40,41^. Testing records, and associated individual demographics (including location of residence), were collected by Rymedi software and provided in excel files.

#### Vaccination records

Full vaccination is defined as being vaccinated with one dose of Ad26.COV2.S or two doses of any other vaccine at least 14 days prior to the prediction start^41^. Individuals are boosted if they received a booster dose of BNT162b2, mRNA-1273 or Ad26.COV2.S at least 7 days prior to the prediction start. Individuals are considered as having no protection from vaccination if they are either unvaccinated or only received one dose of an mRNA vaccine.

During the Fall 2021 semester, the university created a Covid-19 vaccine upload toolkit and provided strong financial incentives to individuals uploading proof of complete vaccination. While data on whether an individual received full vaccination was likely captured with high accuracy^41^, data on the number of individuals with a booster dose is subject to underreporting^9^. Therefore, the fully vaccinated group in the compartment-based modeling framework likely contains a mixed population of fully vaccinated and boosted individuals^9^. Because estimated protection for the fully vaccinated group is based on this population as well, the resulting downstream bias in model prediction is expected to be minimal. We assess sensitivity to this assumption by replacing institutional level estimates of the number of boosted individuals for each population with CDC demographic data of vaccination rates by age group and replace institutional level estimates of protection with literature-based estimates.

#### Isolation/quarantine

Student isolation and quarantine was tracked using a management system, including the software Atlassian Jira^56^. A description for the data application and collection processes are illustrated in McMahan et al. (Figure S1)^57^. Ethical review for this study and obtained by Institutional Review Board of Clemson University (IRB # 2021-043-02).

Additional data sources are provided in Table 2.

**Table 2:** Data sources for the Spring ’22 and Fall ’22 analyses at Clemson University.

Data	Sources	Use
Testing records	Rymedi, Student Health Services, CCIT Test upload toolkit	Surveillance, infection history/protection levels, estimation of disease initial model states and disease transition/transmission parameters, prediction
Vaccination	CCIT vaccination upload toolkit	Protection levels, estimation of initial model states and disease transition/transmission parameters
Isolation/Quarantine	Atlassian Jira	Initial states for isolation/quarantine, effectiveness of isolation/quarantine policies
Demographic and place of residence	CCIT, Rymedi	Estimation of initial model states and disease transition/transmission parameters by subpopulation
Course records	Clemson Records and Registration	Student affiliation status, initial model states
Community characteristics	CDC/US Census	Inform estimates of initial model states and disease transition/transmission parameters for community subpopulation

Testing, vaccination, and demographic data partially come from the Clemson Computing and Information Technology (CCIT). Community characteristics come from the Center for Disease Control (CDC) and US Census Bureau.

### Modeling framework

#### Compartment-based model

We developed a metapopulation compartmental model that projects weekly SARS-CoV-2 cases, symptomatic cases, and daily isolations and quarantines. This model generalizes the metapopulation SEIR model^51^. A diagram of the dynamics across all compartments is presented in Fig. 1.

Each compartment comprises of six sub-populations—in-state residential students, out-of-state residential students, non-residential students, faculty, staff, and community. In addition, each compartment is indexed by $j=0, 1,\dots , 5$, representing each of the following six protection levels:

- $j=0$: unprotected (unvaccinated, no previous infection)
- $j=1$: fully vaccinated without previous infection
- $j=2$: boosted without previous infection
- $j=3$: previously infected, unvaccinated
- $j=4$: fully vaccinated with previous infection
- $j=5$: boosted with previous infection

Within each protection level, individuals are assigned into one of the following compartments at baseline: susceptible individuals (*S*_*j*_), individuals exposed to the disease but not yet infectious (*E*_*j*_), symptomatic (${I}_{{S}_{j}}$) or asymptomatically/mild (${I}_{{A}_{j}}$) infectious, exposed or infectious individuals testing positive (${T}_{{E}_{j}}$ and ${T}_{{I}_{j}}$, respectively), individuals in isolation housing (*H*_*j*_), quarantine for close-contacts of infected individuals who did not contract disease and remain susceptible (${Q}_{{S}_{j}}$), quarantine for close-contacts of infected individuals who were exposed to the disease (${Q}_{{E}_{j}}$), and recovered (*R*_*j*_) for all individuals no longer infectious or susceptible to the disease during the follow-up period. Projections were carried out using the forward Euler method. Each day is divided into six time-steps, four hours each. Details of all model equations of the forward Euler method are provided in Table S1.

Since the five-week projection period is short, we assume that there is no transition from one protection level to another during the projection period. Specifically, there is no transition from unvaccinated to fully vaccinated or from fully vaccinated to boosted. For instance, unprotected susceptible individuals (*S*_*0*_) do not transition into fully vaccinated without previous infection (*S*_*1*_) during the projection period.

In addition, we also assume that symptomatic individuals are voluntarily tested and automatically moved to isolation housing. On the other hand, asymptomatic individuals are only tested under mandatory testing policies. The implication is that under voluntary testing strategy the detected cases are all symptomatic, while under mandatory testing the detected cases include both symptomatic and asymptomatic cases.

#### Transmission

Transmission is governed by the basic reproductive number (*R*_*0*_), contact matrix, and infectivity period. For the no immunity group, *R*_*0*_ is computed by affiliation subpopulation for each SARS-CoV-2 variant based on scientific literature and is internally validated using institutional data. Transmission in the no immunity group is modeled by the parameter *β*_*0*_ = *R*_*0*_ × *ϕ*, where 1/*ϕ* is the infectivity period^58^. For the other immunity groups *j* = 1,2,…,5, the transmission parameter is *β*_*j*_ = *β*_*0*_ × (1 − *hr*_*j*_*)*, where *hr*_*j*_ is the estimated protection for level *j* (estimation discussed in next section). These parameters, along with the contact network matrix, are adjusted to reflect time-dependent changes within and between subpopulations. These time steps correspond to time of day and day of week in order to reflect varying social engagements, including time spent in class, work, and weekends.

*R*_*0*_ for each affiliation in the Spring ’22 analysis is validated using testing data collected during the Fall ’21 semester. Holding all other parameters constant, we searched for the optimal *R*_*0*_ that minimizes the mean squared error between the projected cases and the observed cases in Fall ’21.

#### Estimated protection

In the main analysis (Clemson University Spring ’22), we estimated the protection *r*_*j*_ due to vaccination and/or previous infection using a Cox proportional hazard model. The outcome was the testing results during the pre-arrival testing period prior to semester start between December 31, 2021 and January 9, 2022. Information of vaccination status and previous infections prior to January 9, 2022 was collected from institutional data. To account for the differences between students and employees, we fitted two separate models.

For the *i*th subject, the hazard function is given by

$$h\left(t|{V}_{i},{B}_{i},{P}_{i}\right)={h}_{0}\left(t\right)\times {\text{exp}}\left({a}_{V}\times {V}_{i}+{a}_{B}\times {B}_{i}+{a}_{P}\times {P}_{i}\right),$$

where *V*_*i*_ is an indicator for fully vaccinated without booster, *B*_*i*_ an indicator for boosted, and *P*_*i*_ an indicator for previously infected. Based on preliminary analyses, the interaction between vaccination status and previous infection is not statistically significant (student P-values: ${P}_{V\times P}=0.719$, ${P}_{B\times P}=0.308$; employee P-values: ${P}_{V\times P}=0.157$, ${P}_{B\times P}=0.070$). Hence the effects due to vaccination and due to previous infection are additive.

For protection level *j* = 1, …, 5, the estimated protection is given by 1 − *hr*_*j*_, where *hr*_*j*_ is the hazard ratio relative to the unprotected individuals. Specifically,

1. Fully vaccinated without previous infection: $h{r}_{1}={\text{exp}}({a}_{V})$
2. Boosted without previous infection: $h{r}_{2}={\text{exp}}({a}_{B})$
3. Previously infected without vaccination: $h{r}_{3}={\text{exp}}({a}_{P})$
4. Fully vaccinated with previous infection: $h{r}_{4}={\text{exp}}({a}_{V}+{a}_{P})$
5. Boosted with previous infection: $h{r}_{5}={\text{exp}}({a}_{B}+{a}_{P})$

These estimates for the hazard ratio and the protection level were used in the Spring ’22 analysis for Clemson University, UGA, and PSU. For the Clemson University Fall ’22 analysis, we adopted estimates for the relative risk of infection/reinfection from recent literature, which studied the effect of vaccination and previous infection against the omicron strain.

#### Contact matrix

The interaction among the six subpopulations (in-state residential student, out-of-state residential student, non-residential student, faculty, staff, and community) is modeled via the contact matrix *C*. Individuals in each protection level *j* transition from the susceptible to the exposed compartment at a rate of

$${\beta }_{j}\times C\times \frac{{I}_{tot}}{N},$$

where *I*_*tot*_ is the total number of infectious individuals, *N* is the subpopulation size. Following Lloyd and Jansen (2004), *C* is a $6\times 6$ matrix, where the component *C*_*kl*_ represents the proportion of individuals in subpopulation *k* making contacts with individuals in subpopulation *l* in each time step, with *k, l* = 1, …, 6 denoting subpopulations in the order of in-state residential student, out-of-state residential student, non-residential student, faculty, staff, and community.

To account for different interaction patterns across different time periods of the day and day of the week, the contact matrix *C* assumes different structures during (1) classroom time (weekday, time step 1), (2) work time (weekday, time step 2), (3) after hours (weekday, time step 3–6), and (4) weekend. Full specification of the contact matrix is presented in the Supplementary Appendix 1.

#### Initial model states

Here we give an overview of the estimation procedure for initial model states in the main analysis. Details are provided in the Supplementary Appendix 1. Briefly, the number of currently infected individuals are estimated by the total number of infections within 5-days prior to the follow-up period. Under mandated pre-arrival or arrival testing, infections are divided between the exposed, asymptomatic infectious, and symptomatic infectious compartments. The distribution of infections to each of these compartments is based on the symptomatic infection rate, test sensitivity, and length of the infectivity period for each compartment. The number of individuals in isolation/quarantine is estimated based on the total number of individuals with an exit date from isolation/quarantine after the prediction start date (infected individuals exiting form isolation/quarantine prior to start of follow-up are considered recovered if within 90 days of follow-up).

The recovered compartment consists of all individuals infected between 5 and 90 days prior to follow-up. The Spring 2022 and Fall 2022 analyses are subject to underreporting of both previously infected and recovered compartments due to shifts in university testing strategy (from weekly testing to voluntary testing). To account for underreporting, we estimate the number of unrecorded infections and add them to previously infected compartments (if > 90 days since infection) or recovered (if ≤ 90 days since infection)^40^.

In the community, the proportion of individuals in each protection level is assumed to be the same as the employee subpopulation at Clemson University. Initial values for the testing, isolation and quarantine compartments are all set to 0. The community baseline infection rate, baseline recovery rate, and the proportion of additional recovered individuals can all be customized in the toolkit.

#### Extension to other settings

The estimation of initial states for UGA and PSU has several major differences compared to the main analysis. First, from the university dashboard, we do not have sufficient information of the full vaccination rate, the booster rate, the proportion of the previously infected, or the recently recovered. For other institutions, we estimate the missing information using a combination of data collected by Clemson University and data provided by the Centers for Disease Control and Prevention (CDC). The calculation of subpopulation sizes and other details are provided in the Supplementary Appendix 1. Second, the reported positive cases during the week prior to the prediction start are based on results from voluntary testing, as opposed to mandatory arrival testing in the main analysis based on Clemson University. These cases are assumed to be ${I}_{s}(0)$, the symptomatically infectious at the baseline. The initial in the exposed compartment is given by $E\left(0\right)=\frac{{I}_{s}\left(0\right)}{s{e}_{I}}\times \frac{\sigma }{\gamma }$ and the initial in the asymptotic infection compartment is given by ${I}_{A}\left(0\right)=\frac{{I}_{s}(0)}{s{e}_{I}}\times \frac{\phi }{\gamma }$, where *1/σ*, *1/γ*, and *1/ϕ* are the mean incubation time, mean symptomatic infectious time before isolation, and mean asymptomatic infectious time.

Compared to the main analysis, in the Fall ’22 semester analysis the most notable difference is that the University implemented a voluntary testing strategy in the Fall ’22 semester instead of weekly surveillance testing. Consequently, all baseline infections were assumed to be symptomatic. Due to potential underreporting, potential unreported infections prior to May 23, 2022 (90 days before prediction start) when a voluntary testing policy was in place (December 12, 2021–January 2, 2022; April 2–May 22, 2022) were imputed and added to the previously infected compartments. This is similar to the calculation of additional recovered in the main analysis. In addition, comparing the ratio of Rymedi tests and self-uploaded tests between Summer ’21 and Summer ’22, there was substantial decrease in the self-uploaded testing results in Summer ’22 because of a lack of incentive to do so. We first calculated the additional symptomatic infections in Summer ’22 so that the ratio between self-uploaded results in Summer ’22 matched the results in Summer ’21, and then calculated the asymptomatic infections similar to the additional recovered the main analysis. The total number of additional recovered in Summer ’22 is the additional symptomatic and asymptomatic infections combined.

#### Output metrics

We now describe the output metrics in the Toolkit and the associated statistical methods. The Toolkit displays the projection of the weekly symptomatic SARS-CoV-2 cases and the weekly total cases. The weekly cases are provided in two versions: (1) residential students, non-residential students, faculty, staff; and (2) students, employees.

In addition, the Toolkit also displays the projected daily number of university students and employees in isolation housing or quarantine. The projected isolation and quarantine for students includes numbers for out-of-state residential students, all residential students, and all students.

*Daily and weekly symptomatic cases* Daily symptomatic cases under this framework consist of two groups of individuals, those who are detected at the beginning of the day, and those who are isolated at each time step of the day. Let $\Delta$ be the time step in hours and $h=\Delta /24$ be the time step in days, so that ${h}^{-1}=24/\Delta$ is the number of time steps per day. The number of new symptomatic cases on day *t* is

1 $${I}_{s}\left(t\right)=\sum_{j=0}^{5}{I}_{{s}_{j}}\left(t\right)\times p\times s{e}_{I}+\sum_{k=0}^{{h}^{-1}-1}\sum_{j=0}^{5}{I}_{{s}_{j}}\left(t+kh\right)\times \gamma \times h,$$

where *p* is the daily testing proportion, *se*_*I*_ is the testing sensitivity for symptomatic infections, and *1/γ* is the mean time of symptomatic infection before isolation. Weekly symptomatic cases are computed by aggregating the daily symptomatic cases over 7 days.

*Daily and weekly detected cases* Daily detected cases include the daily symptomatic cases in Eq. (1), the daily detected asymptomatic cases, and the daily detected exposed individuals. The number of new detected cases on day *t* is

2 $$D\left(t\right)={I}_{s}\left(t\right)+\sum_{j=0}^{5}({I}_{{a}_{j}}\left(t\right)\times p\times s{e}_{I}+{E}_{j}\left(t\right)\times p\times s{e}_{E}),$$

where *se*_*E*_ is the testing sensitivity for the exposed individuals. Weekly detected cases are computed by aggregating the daily detected cases over 7 days.

*Total cases* Daily new cases on each day are calculated via the difference in the susceptible compartments between day *t-1* and *t.* The number of new cases on day *t* is given by

3 $$Total\left(t\right)=\sum_{j=0}^{5}\left[{S}_{j}\left(t-1\right)+{Q}_{{s}_{j}}\left(t-1\right)\right]-\sum_{j=0}^{5}\left[{S}_{j}\left(t\right)+{Q}_{{s}_{j}}\left(t\right)\right].$$

Weekly new cases aggregate daily new infections over 7 days. Note that the total cases include both detected and undetected cases.

*Daily isolation* The number of isolations on day *t* is the total number of individuals in all isolation compartments, i.e., $H\left(t\right)=\sum_{j=0}^{5}{H}_{j}(t)$.

*Daily isolation and quarantine* The number of isolations and quarantine on day *t* is the number of individuals in all isolation/quarantine compartments, i.e., $IQ\left(t\right)=\sum_{j=0}^{5}\left[{H}_{j}\left(t\right)+{Q}_{{s}_{j}}\left(t\right)+{Q}_{{E}_{j}}\left(t\right)\right]$.

### Supplementary Information

Supplementary Information.

## Data Availability

All data and R code needed to reproduce the conclusions of this paper are present in the Supplementary Materials. Data and code for this work, including the publicly available toolkit, can be accessed in the following links: https://github.com/ZichenM/CampusPredictionApp and https://zmstats.shinyapps.io/CampusPrediction/. Requests for additional aggregated, de-identified data related to this study should be submitted to L. Rennert (liorr@clemson.edu).
