# Supplementary material for: An epidemiological modeling framework to inform institutional-level response to infectious disease outbreaks: a Covid-19 case study
Source: Sci Rep. 2024 Mar 27;14:7221. doi: 10.1038/s41598-024-57488-y (PMC10973339; doi:10.1038/s41598-024-57488-y)
Supplement: Supplementary file 1 — Supplementary Information. [file 41598_2024_57488_MOESM1_ESM.docx]

Supplementary Materials for

**An epidemiological modeling framework to inform institutional-level response to infectious disease outbreaks: A Covid-19 case study**

Zichen Ma and Lior Rennert*

*Corresponding author. Email: [liorr@clemson.edu](mailto:liorr@clemson.edu)

**This file includes:**

Supplementary Text

Fig. S1

Tables S1 to S16

**SUPPLEMENTARY TEXT**

Supplementary Appendix 1. Metapopulation Compartmental Model

We developed a metapopulation compartmental model that projects weekly SARS-CoV-2 cases, symptomatic cases, and daily isolations and quarantines. This model generalizes the metapopulation SEIR model.^1^

As described in the *Methods* section of the manuscript, each compartment comprises of six sub-populations, including in-state residential students, out-of-state residential students, non-residential students, faculty, staff, and community. In addition, each compartment is indexed by $j=0, 1,\ldots, 5$, representing each of the following six protection levels:

- $j=0$: unprotected (unvaccinated, no previous infection)
- $j=1$: fully vaccinated without previous infection
- $j=2$: boosted without previous infection
- $j=3$: previously infected, unvaccinated
- $j=4$: fully vaccinated with previous infection
- $j=5$: boosted with previous infection

Within each protection level, individuals who have not been exposed are susceptible ($S_{j}$). The $E_{j}$ compartment consists of individuals who are exposed to the disease but not yet infectious. The transmission rate from $S_{j}$ to $E_{j}$ varies across different protection levels. After an incubation period, exposed individuals become either symptomatic ($I_{S_{j}}$) or asymptomatic ($I_{A_{j}}$). To distinguish the testing sensitivity between exposed and infectious individuals, the model assumes two different compartments for testing: $T_{I_{j}}$ includes infectious individuals who test positive, and $T_{E_{j}}$ includes exposed individuals who test positive. After a test turnaround time of 1 day, individuals with a positive test are moved to isolation housing ($H_{j}$). In addition, the model also assumes that symptomatically infectious individuals voluntarily get tested within 2 days of developing symptoms and are transferred to isolation housing. Close-contacts to the infected are moved to two different quarantine compartments – $Q_{S_{j}}$ for those who did not contract the disease, and $Q_{E_{j}}$ for those that did contract the disease. Following the isolation or quarantine period, individuals in $Q_{S_{j}}$ transfer back to $S_{j}$ and become susceptible again, while individuals in $H_{j}$ or $Q_{E_{j}}$ move to the recovered compartment ($R_{j}$). A list of model equations is provided in Table S1. Initial states for each compartment are provided in Tables S2-S5. Model input parameters are provided in Tables S6-S8.

In the rest of this section, we detail the initial states of the compartments and the statistical methods to calculate the input parameters.

**1. Initial states.** The *Results* section of the manuscript presented projections in three different scenarios. Initial states of various compartments were calculated differently in each scenario due to the availability of data, different testing protocols, and potential underreporting of infections. Here we discuss them separately.

*Clemson University – Spring ’22*

In the first scenario, we projected the overall SARS-CoV-2 cases and symptomatic cases for the first five weeks of the Spring ’22 semester at Clemson University from January 10 to February 13. During this period, the University implemented a mandatory weekly testing protocol for all students and employees.

1. **Population size (**$\boldsymbol{N}_{\boldsymbol{j}}$**).** $N_{j}$ is a vector of length six, in the order of in-state residential students, out-of-state residential students, non-residential students, faculty, staff, and community, representing the total number of individuals in each sub-population with protection level $j$. For Clemson students and employees,

- $N_{0}:$ Individuals who were unprotected, i.e., no reported vaccination or previous infections.
- $N_{1}:$ Individuals who were fully vaccinated without previous infections. Full vaccination includes receiving one dose of Ad26.COV2.S or two doses of any other vaccine at least 14 days prior to the prediction start (January 10, 2022).
- $N_{2}$: Individuals who were boosted without previous infections. Booster includes receiving a booster dose of BNT162b2, mRNA-1273 or Ad26.COV2.S at least 7 days prior to the prediction start.

Due to potential underreporting of the booster shot, we adjust $N_{1}$ and $N_{2}$ according to the estimated proportion of boosted among fully vaccinated from the Center for Disease Control and Prevention (CDC):

$$N_{1}^{adj.}=\left( 1-p^{booster} \right)\left( N_{1}^{obs.}+N_{2}^{obs.} \right),$$

( 1 )

$$N_{2}^{adj.}=p^{booster}\left( N_{1}^{obs.}+N_{2}^{obs.} \right),$$

( 2 )

where $p^{booster}$ is the proportion of boosted individuals among fully vaccinated according to the CDC, $N_{1}^{obs.}$ and $N_{2}^{obs.}$ are the reported fully vaccinated and boosted individuals at Clemson University.

- $N_{3}$: Individuals who were previously infected without vaccination. Previous infection is defined as testing positive prior to October 12, 2021, 90 days prior to January 10, 2022.
- $N_{4}$: Individuals who were fully vaccinated and previously infected. This is similar to $N_{1}$ but with previous infection.
- $N_{5}$: Individuals who were boosted and previously infected. This is similar to $N_{2}$ but with previous infection.

Here we make another adjustment to $N_{4}$ and $N_{5}$ to account for the potential underreporting of boosters:

$$N_{4}^{adj.}=\left( 1-p^{booster} \right)\left( N_{4}^{obs.}+N_{5}^{obs.} \right),$$

( 3 )

$$N_{5}^{adj.}=p^{booster}\left( N_{4}^{obs.}+N_{5}^{obs.} \right),$$

( 4 )

For the community sub-population, the 17,681 residents in the City of Clemson^2^ are distributed to $N_{0}, N_{1}, \ldots, N_{5}$ according to the distribution of Clemson employees.

1. **Recovered (**$\boldsymbol{R}_{\boldsymbol{j}}\boldsymbol{(0)}$**).** Recently recovered individuals at time 0 are individuals who tested positive between 5 and 90 days prior to January 10, 2022.
   1. **Additional recovered.** Clemson University switched from mandatory testing in Fall ’21 to voluntary testing between December 12, 2021 and January 2, 2022, during which students and employees were not required to test weekly. To account for the underreporting of infections during this period, an additinal fraction of individuals in $N_{j}$ are added to $R_{j}(0)$ as additional recovered. The adjusted $R_{j}(0)$ is

$$R_{j}^{adj.}\left( 0 \right)=R_{j}^{obs.}\left( 0 \right)+R_{j}^{vol.}\frac{1-\alpha}{\alpha}=R_{j}^{obs.}\left( 0 \right)+\frac{R_{j}^{vol.}\frac{1-\alpha}{\alpha}}{N_{j}}\times N_{j},$$

( 5 )

where $R_{j}^{obs.}(0)$ is the overall observed recovered individuals, $R_{j}^{vol.}$ is the individuals tested positive during the voluntary testing period between Dec. 12, 2021 and Jan. 2, 2022, and $\alpha$ is the proportion of individuals detected through voluntary testing. This adjustment was calculated separately for students and employees due to the different values for $\alpha$ across different sub-populations. In the Toolkit, we set the proportion of additional recovered, $p_{j}^{ar}$, in each sub-population as

$$p_{j}^{ar}=\frac{R_{j}^{vol.}\frac{1-\alpha}{\alpha}}{N_{j}}.$$

( 6 )

1. **Exposed, symptomatic/asymptomatic infectious (**$\boldsymbol{E}_{\boldsymbol{j}}\left( \boldsymbol{0} \right)\boldsymbol{,}\boldsymbol{I}_{\boldsymbol{S}_{\boldsymbol{j}}}\left( \boldsymbol{0} \right)\boldsymbol{,}\boldsymbol{I}_{\boldsymbol{A}_{\boldsymbol{j}}}\boldsymbol{(0)}$**).** From empirical data collected at Clemson University under mandatory testing between Jan. 6 and Jan. 9, 2022, we obtained the total number of individuals who tested positive, $I_{j}^{tot}$. The initial states for exposed, symptomatic infectious, and asymptomatic infectious compartments were calculated according to

$$E_{j}\left( 0 \right)=I_{j}^{tot}\frac{\sigma}{\sigma+\gamma+\phi}\cdot\frac{1}{se_{E}},$$

$$I_{S_{j}}\left( 0 \right)=I_{j}^{tot}\frac{\gamma}{\sigma+\gamma+\phi}\cdot\frac{1}{se_{I}},$$

$$I_{A_{j}}\left( 0 \right)=I_{j}^{tot}\frac{\phi}{\sigma+\gamma+\phi}\cdot\frac{1}{se_{I}},$$

( 7 )

where $1/\sigma$, $1/\gamma$, and $1/\phi$ are the mean incubation time, mean symptomatic infectious time, and mean asymptomatic infectious time before detection/isolation, and $se_{E}$ and $se_{I}$ are the testing sensitivity for exposed and infectious individuals, respectively.

1. **Isolation housing and quarantine (**$\boldsymbol{H}_{\boldsymbol{j}}\left( \boldsymbol{0} \right)\boldsymbol{,}\boldsymbol{Q}_{\boldsymbol{E}_{\boldsymbol{j}}}\left( \boldsymbol{0} \right)\boldsymbol{,}\boldsymbol{Q}_{\boldsymbol{S}_{\boldsymbol{j}}}\boldsymbol{(0)}$**).** $H_{j}(0)$ consists of individuals who were isolated as of Jan. 10, 2022. The observed individuals under quarantine as of Jan. 10, 2022 were $Q_{E_{j}}\left( 0 \right)+Q_{S_{j}}(0)$, where

$$Q_{E_{j}}\left( 0 \right)=\frac{I_{j}^{tot}}{N_{j}}\left[ Q_{E_{j}}\left( 0 \right)+Q_{S_{j}}\left( 0 \right) \right]$$

( 8 )

included quarantined individuals who contracted the disease, and

$$Q_{S_{j}}\left( 0 \right)=\left( 1-\frac{I_{j}^{tot}}{N_{j}} \right)\left[ Q_{E_{j}}\left( 0 \right)+Q_{S_{j}}\left( 0 \right) \right]$$

( 9 )

included quarantined individuals who did not contract the disease.

1. **Test positive (**$\boldsymbol{T}_{\boldsymbol{I}_{\boldsymbol{j}}}\left( \boldsymbol{0} \right)\boldsymbol{,}\boldsymbol{T}_{\boldsymbol{E}_{\boldsymbol{j}}}\boldsymbol{(0)}$**).** The initial states for these compartments were set to 0.
2. **Susceptible (**$\boldsymbol{S}_{\boldsymbol{j}}\boldsymbol{(0)}$**).** All individuals not included in the other compartments at the baseline were considered susceptible:

$$S_{j}\left( 0 \right)=N_{j}-E_{j}\left( 0 \right)-I_{S_{j}}\left( 0 \right)-I_{A_{j}}\left( 0 \right)-H_{j}\left( 0 \right)-Q_{E_{j}}\left( 0 \right)-Q_{S_{j}}\left( 0 \right)-R_{j}\left( 0 \right).$$

( 10 )

*UGA and PSU – Spring ’22*

In the second analysis, we made projections on the number of cases for the first five weeks of the Spring ’22 semester at the University of Georgia (UGA) and Pennsylvania State University (PSU). The projection time frame was January 10 to February 13, 2022, the same as in the Clemson analysis. Initial states in the two analyses for UGA and PSU were largely imputed from the initial states in the Clemson University Spring ’22 analysis. Listed below are the differences.

1. **Population size (**$\boldsymbol{N}_{\boldsymbol{j}}$**).** From the website of UGA and PSU, we obtained the total number of students and employees in each of the two institutions. These numbers were then distributed to different sub-populations and protection levels proportional to the sub-population sizes at Clemson University.
2. **Recovered (**$\boldsymbol{R}_{\boldsymbol{j}}\boldsymbol{(0)}$**).** Unadjusted recovered individuals in each protection level were proportional to the unadjusted recovered individuals at Clemson University,

$$R_{j}\left( 0 \right)=N_{j}\frac{R_{j}^{CU}(0)}{N_{j}^{CU}},$$

( 11 )

where $R_{j}^{CU}(0)$ is the unadjusted recovered and $N_{j}^{CU}$ is the population size at Clemson University. The proportion of additional recovered, $p_{j}^{ar}$, was estimated from the Clemson University Spring ’22 analysis.

1. **Exposed, symptomatic/asymptomatic infectious (**$\boldsymbol{E}_{\boldsymbol{j}}\left( \boldsymbol{0} \right)\boldsymbol{,}\boldsymbol{I}_{\boldsymbol{S}_{\boldsymbol{j}}}\left( \boldsymbol{0} \right)\boldsymbol{,}\boldsymbol{I}_{\boldsymbol{A}_{\boldsymbol{j}}}\boldsymbol{(0)}$**).** From the Covid Dashboard at UGA and PSU, the two institutions were under a voluntary testing protocol during the week prior to semester start. The total number of detected infections during the week prior to prediction start, $I_{tot}$, were assumed to be symptomatic and the initial state of $I_{S_{j}}(0)$ was given by

$$I_{S_{j}}\left( 0 \right)=\frac{I_{tot}}{se_{I}}.$$

( 12 )

For exposed and asymptomatic infection compartments,

$$E_{j}\left( 0 \right)=I_{tot}\frac{\sigma}{\gamma}\cdot\frac{1}{se_{E}},$$

$$I_{A_{j}}\left( 0 \right)=I_{tot}\frac{\phi}{\gamma}\cdot\frac{1}{se_{I}}.$$

( 13 )

1. **Isolation housing and quarantine (**$\boldsymbol{H}_{\boldsymbol{j}}\left( \boldsymbol{0} \right)\boldsymbol{,}\boldsymbol{Q}_{\boldsymbol{E}_{\boldsymbol{j}}}\left( \boldsymbol{0} \right)\boldsymbol{,}\boldsymbol{Q}_{\boldsymbol{S}_{\boldsymbol{j}}}\boldsymbol{(0)}$**).** Numbers of individuals under isolation and quarantine at the start of the prediction were not reported on the Dashboard, hence calculated proportional to the observed numbers in the Clemson Spring ’22 analysis.

$$H_{j}\left( 0 \right)=N_{j}\frac{H_{j}^{CU}(0)}{N_{j}^{CU}},$$

$$Q_{E_{j}}\left( 0 \right)=N_{j}\frac{Q_{E_{j}}^{CU}(0)}{N_{j}^{CU}},$$

$$Q_{S_{j}}\left( 0 \right)=N_{j}\frac{Q_{S_{j}}^{CU}(0)}{N_{j}^{CU}}.$$

( 14 )

1. **Test positive (**$\boldsymbol{T}_{\boldsymbol{I}_{\boldsymbol{j}}}\left( \boldsymbol{0} \right)\boldsymbol{,}\boldsymbol{T}_{\boldsymbol{E}_{\boldsymbol{j}}}\boldsymbol{(0)}$**).** The initial states for these compartments were set to 0.
2. **Susceptible (**$\boldsymbol{S}_{\boldsymbol{j}}\boldsymbol{(0)}$**).** All individuals not included in the other compartments at the baseline were considered susceptible:

$$S_{j}\left( 0 \right)=N_{j}-E_{j}\left( 0 \right)-I_{S_{j}}\left( 0 \right)-I_{A_{j}}\left( 0 \right)-H_{j}\left( 0 \right)-Q_{E_{j}}\left( 0 \right)-Q_{S_{j}}\left( 0 \right)-R_{j}\left( 0 \right).$$

( 15 )

*Clemson University – Fall ’22*

In the last analysis, we projected the number of cases for the first five weeks of the Fall ’22 semester at Clemson University between August 24 and September 27, 2022. The population consists of all active students and employees at the start of Fall ’22 as well as the local residents in the community at large. Compared to the Spring ’22 analysis, the main difference is that the University implemented a voluntary testing protocol in Fall ’22 instead of a mandatory testing protocol, which impacted the population size of previously infected as well as the initial of the recently recovered.

1. **Population size (**$\boldsymbol{N}_{\boldsymbol{j}}$**).** The unadjusted population sizes are obtained from empirical data at Clemson University. These numbers were adjusted to account for the potential underreporting of infections between December 12, 2021 and January 2, 2022. The potentially underreported individuals were moved from uninfected sub-populations to a corresponding sub-population for the previously infected while keeping the vaccination status the same. Specifically,

$$N_{0}^{adj.}=N_{0}^{obs.}-N_{3}^{vol.}\frac{1-\alpha}{\alpha},$$

$$N_{3}^{adj.}=N_{3}^{obs.}+N_{3}^{vol.}\frac{1-\alpha}{\alpha},$$

$$N_{1}^{adj.}=N_{1}^{obs.}-N_{4}^{vol.}\frac{1-\alpha}{\alpha},$$

$$N_{4}^{adj.}=N_{4}^{obs.}+N_{4}^{vol.}\frac{1-\alpha}{\alpha},$$

$$N_{2}^{adj.}=N_{2}^{obs.}-N_{5}^{vol.}\frac{1-\alpha}{\alpha},$$

$$N_{5}^{adj.}=N_{5}^{obs.}+N_{5}^{vol.}\frac{1-\alpha}{\alpha},$$

( 16 )

where $N_{j}^{vol.}$ represents the observed number of individuals who tested positive between Dec. 12, 2021 and Jan. 2, 2022, and $N_{j}^{obs.}$ represents the observed population size.

1. **Recovered (**$\boldsymbol{R}_{\boldsymbol{j}}\boldsymbol{(0)}$**).** Unadjusted recovered individuals were obtained from empirical data, consisting of those who tested positive between May 26 and August 19, 2022.
2. **Exposed, symptomatic/asymptomatic infectious (**$\boldsymbol{E}_{\boldsymbol{j}}\left( \boldsymbol{0} \right)\boldsymbol{,}\boldsymbol{I}_{\boldsymbol{S}_{\boldsymbol{j}}}\left( \boldsymbol{0} \right)\boldsymbol{,}\boldsymbol{I}_{\boldsymbol{A}_{\boldsymbol{j}}}\boldsymbol{(0)}$**).** Similar to the UGA and PSU analysis, the total number of detected infections during the week prior to prediction start, $I_{tot}$, were assumed to be symptomatic and the initial state of $I_{S_{j}}(0)$ was given by

$$I_{S_{j}}\left( 0 \right)=\frac{I_{tot}}{se_{I}}.$$

( 17 )

For exposed and asymptomatic infection compartments,

$$E_{j}\left( 0 \right)=I_{tot}\frac{\sigma}{\gamma}\cdot\frac{1}{se_{E}},$$

$$I_{A_{j}}\left( 0 \right)=I_{tot}\frac{\phi}{\gamma}\cdot\frac{1}{se_{I}}.$$

( 18 )

1. **Isolation housing and quarantine (**$\boldsymbol{H}_{\boldsymbol{j}}\left( \boldsymbol{0} \right)\boldsymbol{,}\boldsymbol{Q}_{\boldsymbol{E}_{\boldsymbol{j}}}\left( \boldsymbol{0} \right)\boldsymbol{,}\boldsymbol{Q}_{\boldsymbol{S}_{\boldsymbol{j}}}\boldsymbol{(0)}$**).** The numbers of individuals under isolation and quarantine were obtained using empirical data. The distribution of quarantine numbers in to $Q_{E_{j}}(0)$ and $Q_{S_{j}}(0)$ compartments was calculated the same as in the Clemson Spring ’22 analysis.
2. **Test positive (**$\boldsymbol{T}_{\boldsymbol{I}_{\boldsymbol{j}}}\left( \boldsymbol{0} \right)\boldsymbol{,}\boldsymbol{T}_{\boldsymbol{E}_{\boldsymbol{j}}}\boldsymbol{(0)}$**).** The initial states for these compartments were set to 0.
3. **Susceptible (**$\boldsymbol{S}_{\boldsymbol{j}}\boldsymbol{(0)}$**).** All individuals not included in the other compartments at the baseline were considered susceptible:

$$S_{j}\left( 0 \right)=N_{j}-E_{j}\left( 0 \right)-I_{S_{j}}\left( 0 \right)-I_{A_{j}}\left( 0 \right)-H_{j}\left( 0 \right)-Q_{E_{j}}\left( 0 \right)-Q_{S_{j}}\left( 0 \right)-R_{j}\left( 0 \right).$$

( 19 )

**2. Estimation of protection from vaccination and previous infection.** We now detail the calculation of the protection parameter for individuals with vaccination or previous infection.

In the Clemson University Spring ’22 analysis, we estimated the protection from vaccination and previous infection using a Cox proportional hazard model based on data collected at Clemson University within 10 days from the prediction start between December 31, 2021 and January 9, 2022. To account for the differences between students, who were primarily young adults, and employees, we fitted separate models for students and employees.

For the *i^th^* subject, the hazard function is given by

$$h\left( t|V_{i},B_{i},P_{i} \right)=h_{0}\left( t \right)\exp\left( a_{V}V_{i}+a_{B}B_{i}+a_{P}P_{i} \right),$$

( 20 )

where *V_i_* is an indicator for fully vaccinated without booster, *B_i_* an indicator for boosted, and *P_i_* an indicator for previously infected. Based on preliminary analyses, there is no significant interaction between vaccination status and previous infection. Hence the effects due to vaccination and due to previous infection are additive.

For protection level *j = 1, …, 5*, the estimated protection is given by *1 - hr_j_*, where *hr_j_* is the hazard ratio relative to the unprotected individuals. Specifically,

1. Fully vaccinated without previous infection: $hr_{1}=exp(a_{V})$
2. Boosted without previous infection: $hr_{2}=exp(a_{B})$
3. Previously infected without vaccination: $hr_{3}=exp(a_{P})$
4. Fully vaccinated with previous infection: $hr_{4}=exp(a_{V}+a_{P})$
5. Boosted with previous infection: $hr_{5}=exp(a_{B}+a_{P})$

These estimates for the protection and the hazard ratio were also used in the Spring ’22 analysis for UGA and PSU. For the Clemson University Fall ’22 analysis, we adopted estimates for the relative risk of infection/reinfection from recent literature, which studied the effect of vaccination and previous infection against the omicron strain.

**3. Reproduction number.** The basic reproduction number for each meta-population was validated using data from the Fall 2021 semester at Clemson University. The estimation procedure utilized a grid search across values of the reproduction number minimizing the sum of squares between compartmental model predicted infections and observed infections. The resulting values for residential students, non-residential students, faculty, and staff were 6.0, 5.0, 2.5, and 3.25, respectively. An assumption was made to set community level transmission equal to that of staff. Using data from early studies estimated a plausible range of values for the relative transmission of Omicron to background variants,^3^ we multiplied the estimated reproductive numbers estimated for the Delta variant by the constant 1.67. We note however that results from later studies obtained after our projections estimated this constant in the range of 1.88 to 2.19.^4^

**4. Transmission rate.** The transmission rate for unprotected individuals, $\beta_{0}$, is given by

$$\beta_{0}=R_{0}\phi,$$

( 21 )

where $R_{0}$ is the basic reproduction number, and $1/\phi$ is the mean asymptomatic infection time. The basic reproduction number was estimated and validated using empirical data at Clemson University in the Fall ’21 semester and adjusted to the omicron strain according to existing literature.

For protection level $j=1, 2,\ldots, 5,$ the transmission rate $\beta_{j}$ is adjusted according to $\beta_{j}=\beta_{0}\times hr_{j}$, where $hr_{j}$ is the estimated hazard ratio for level $j$.

**5. Contact matrix.** Individuals in each protection level $j$ transition from the susceptible to the exposed compartment at a rate of

$$\beta_{j}C\frac{I_{tot}}{N},$$

( 22 )

where $I_{tot}$ is the total number of infectious individuals, $N$ is the subpopulation size, and $C$ is the contact matrix that models the interaction across different subpopulations. Following Lloyd and Jansen (2004), the contact matrix $C$ is a $6\times6$ matrix, where the component $C_{kl}$ is defined as the proportion of individuals in subpopulation $k$ that have made contacts with individuals in subpopulation $l$, with $k,l=1,2,\ldots,6,$ denoting subpopulations in the order of in-state residential student, out-of-state residential student, non-residential student, faculty, staff, or community. For each row $k$,

$$\sum_{l=1}^{6} C_{kl}=1.$$

( 23 )

Further, in the model, each day is divided into six time-steps, each representing four hours. To account for the different interaction patterns across subpopulations during different time steps on weekdays (Monday through Friday) as well as on weekends, the contact matrix assumes different values as follows.

1. Weekday, time step 1: We assume that this time step corresponds to classroom time. The contact matrix is given by

$$C=\left( \begin{matrix} 0.55p_{r} & 0.55(1-p_{r}) & \begin{matrix} 0.30 & 0.10 & \begin{matrix} 0.05 & 0.00 \end{matrix} \end{matrix} \\ 0.55p_{r} & 0.55(1-p_{r}) & \begin{matrix} 0.30 & 0.10 & \begin{matrix} 0.05 & 0.00 \end{matrix} \end{matrix} \\ \begin{matrix} 0.30p_{r} \\ 0.30p_{r} \\ \begin{matrix} 0.10p_{r} \\ 0.00 \end{matrix} \end{matrix} & \begin{matrix} 0.30(1-p_{r}) \\ 0.30(1-p_{r}) \\ \begin{matrix} 0.10(1-p_{r}) \\ 0.00 \end{matrix} \end{matrix} & \begin{matrix} \begin{matrix} 0.55 & 0.10 & \begin{matrix} 0.05 & 0.00 \end{matrix} \end{matrix} \\ \begin{matrix} 0.60 & 0.05 & \begin{matrix} 0.05 & 0.00 \end{matrix} \end{matrix} \\ \begin{matrix} \begin{matrix} 0.20 \\ 0.00 \end{matrix} & \begin{matrix} 0.10 \\ 0.00 \end{matrix} & \begin{matrix} \begin{matrix} 0.60 & 0.00 \end{matrix} \\ \begin{matrix} 0.00 & 1.00 \end{matrix} \end{matrix} \end{matrix} \end{matrix} \end{matrix} \right),$$

( 24 )

where $p_{r}$ is the proportion of residential students that are in-state, estimated using data from Clemson University. For this time step, we also adjust the transmission parameter $\beta_{0}$ according to

$$\beta_{0}=\left( 0.5,0.5,0.5,0.5,1,1 \right)R_{0}\phi.$$

( 25 )

1. Weekday, time step 2: We assume that this time step corresponds to work time not inside classrooms. The contact matrix is given by

$$C=\left( \begin{matrix} 0.80p_{r} & 0.80(1-p_{r}) & \begin{matrix} 0.10 & 0.05 & \begin{matrix} 0.05 & 0.00 \end{matrix} \end{matrix} \\ 0.80p_{r} & 0.80(1-p_{r}) & \begin{matrix} 0.10 & 0.05 & \begin{matrix} 0.05 & 0.00 \end{matrix} \end{matrix} \\ \begin{matrix} 0.10p_{r} \\ 0.10p_{r} \\ \begin{matrix} 0.10p_{r} \\ 0.00 \end{matrix} \end{matrix} & \begin{matrix} 0.10(1-p_{r}) \\ 0.10(1-p_{r}) \\ \begin{matrix} 0.10(1-p_{r}) \\ 0.00 \end{matrix} \end{matrix} & \begin{matrix} \begin{matrix} 0.80 & 0.05 & \begin{matrix} 0.05 & 0.00 \end{matrix} \end{matrix} \\ \begin{matrix} 0.20 & 0.60 & \begin{matrix} 0.10 & 0.00 \end{matrix} \end{matrix} \\ \begin{matrix} \begin{matrix} 0.20 \\ 0.00 \end{matrix} & \begin{matrix} 0.10 \\ 0.00 \end{matrix} & \begin{matrix} \begin{matrix} 0.60 & 0.00 \end{matrix} \\ \begin{matrix} 0.00 & 1.00 \end{matrix} \end{matrix} \end{matrix} \end{matrix} \end{matrix} \right).$$

( 26 )

The transmission parameter $\beta_{0}$ is given by

$$\beta_{0}=\left( 0.9,0.9,0.9,1,1,1 \right)R_{0}\phi.$$

( 27 )

1. Weekday, time step 3-6: Time steps 3-6 represent after hours on weekdays. The contact matrix is given by

$$C=\left( \begin{matrix} 0.95p_{r} & 0.95(1-p_{r}) & \begin{matrix} 0.05 & 0.00 & \begin{matrix} 0.00 & 0.00 \end{matrix} \end{matrix} \\ 0.95p_{r} & 0.95(1-p_{r}) & \begin{matrix} 0.05 & 0.00 & \begin{matrix} 0.00 & 0.00 \end{matrix} \end{matrix} \\ \begin{matrix} 0.05p_{r} \\ 0.00 \\ \begin{matrix} 0.00 \\ 0.02 \end{matrix} \end{matrix} & \begin{matrix} 0.05(1-p_{r}) \\ 0.00 \\ \begin{matrix} 0.00 \\ 0.02 \end{matrix} \end{matrix} & \begin{matrix} \begin{matrix} 0.95 & 0.00 & \begin{matrix} 0.00 & 0.00 \end{matrix} \end{matrix} \\ \begin{matrix} 0.00 & 0.75 & \begin{matrix} 0.00 & 0.25 \end{matrix} \end{matrix} \\ \begin{matrix} \begin{matrix} 0.00 \\ 0.02 \end{matrix} & \begin{matrix} 0.00 \\ 0.02 \end{matrix} & \begin{matrix} \begin{matrix} 0.75 & 0.25 \end{matrix} \\ \begin{matrix} 0.02 & 0.92 \end{matrix} \end{matrix} \end{matrix} \end{matrix} \end{matrix} \right).$$

( 28 )

The transmission parameter $\beta_{0}$ is assumed to be the *reference level,* given by

$$\beta_{0}=R_{0}\phi.$$

( 29 )

1. Weekend: All time steps during the weekend have the same contact matrix

$$C=\left( \begin{matrix} 0.85p_{r} & 0.85(1-p_{r}) & \begin{matrix} 0.10 & 0.00 & \begin{matrix} 0.00 & 0.05 \end{matrix} \end{matrix} \\ 0.85p_{r} & 0.85(1-p_{r}) & \begin{matrix} 0.10 & 0.00 & \begin{matrix} 0.00 & 0.05 \end{matrix} \end{matrix} \\ \begin{matrix} 0.05p_{r} \\ 0.00 \\ \begin{matrix} 0.00 \\ 0.03 \end{matrix} \end{matrix} & \begin{matrix} 0.05(1-p_{r}) \\ 0.00 \\ \begin{matrix} 0.00 \\ 0.03 \end{matrix} \end{matrix} & \begin{matrix} \begin{matrix} 0.85 & 0.00 & \begin{matrix} 0.00 & 0.10 \end{matrix} \end{matrix} \\ \begin{matrix} 0.00 & 0.50 & \begin{matrix} 0.00 & 0.50 \end{matrix} \end{matrix} \\ \begin{matrix} \begin{matrix} 0.00 \\ 0.03 \end{matrix} & \begin{matrix} 0.00 \\ 0.03 \end{matrix} & \begin{matrix} \begin{matrix} 0.50 & 0.50 \end{matrix} \\ \begin{matrix} 0.03 & 0.88 \end{matrix} \end{matrix} \end{matrix} \end{matrix} \end{matrix} \right).$$

( 30 )

The transmission parameter $\beta_{0}$ is given by

$$\beta_{0}=\left( 2,2,2,1.5,1.5,1.5 \right)R_{0}\phi.$$

( 31 )

Note that in the above, $\beta_{0}$ represents the transmission rate among unprotected individuals *per time step*.

**SUPPLEMENTARY FIGURES**

**Figure S1.**

**Comparison of predicted cases under different testing strategies at Clemson University in Fall ’22.**


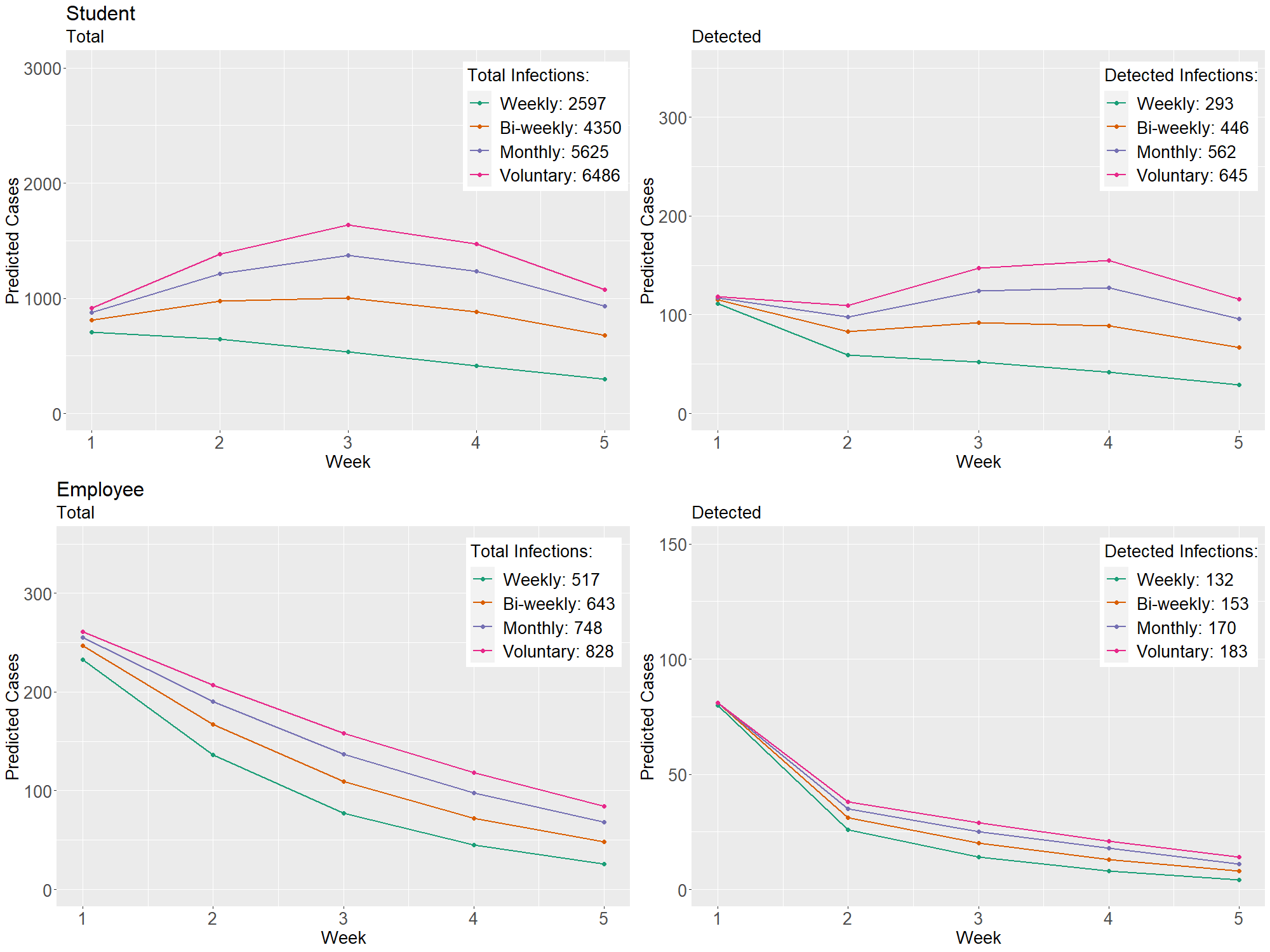


**Fig. S1.** Comparison of predicted cases under different testing strategies at Clemson University during first 5 weeks of Fall ’22 (August 24^th^ through September 27^th^, 2022).

**SUPPLEMENTARY TABLES**

**Table S1.**

**Equations for compartmental transmission models.** Time *t* increases from 0 to *T* days with increments of *h = Δ/24 days with Δ=4 being the increments in hours*. All compartments are 6-dimensional vectors, representing in-state on-campus students, out-of-state on-campus students, off-campus students, faculty, staff, and community numbers in this order.

**Table S1. Equations for compartmental transmission models.** Time *t* increases from 0 to *T* days with increments of *h = Δ/24 days with Δ=4 being the increments in hours*. All compartments are 6-dimensional vectors, representing in-state on-campus students, out-of-state on-campus students, off-campus students, faculty, staff, and community numbers in this order.

| **Compartment** | **Equation** |
| --- | --- |
| **Unprotected** |  |
| …Susceptible: $S_{0}$ | $S_{0}\left( t+h \right)=S_{0}\left( t \right)+\left[ \rho_{Q_{S}}\times Q_{s_{0}}\left( t \right)-\beta_{0}\times C\times I_{tot}\times\frac{S_{0}\left( t \right)}{N} \right]\times h$ |
| …Exposed: $E_{0}$ | $E_{0}\left( t+h \right)=E_{0}\left( t \right)+\left[ \beta_{0}\times C\times I_{tot}\times\frac{S_{0}\left( t \right)}{N}-\sigma\times E_{0}(t) \right]\times h$ |
| …Asymptomatic infectious: $I_{A_{0}}$ | $I_{A_{0}}\left( t+h \right)=I_{A_{0}}\left( t \right)+\left[ \left( 1-\alpha\right)\times\sigma\times E_{0}\left( t \right)-\phi\times I_{A_{0}}(t) \right]\times h$ |
| …Symptomatic infectious: $I_{S_{0}}$ | $I_{S_{0}}\left( t+h \right)=I_{S_{0}}\left( t \right)+\left[ \alpha\times\sigma\times E_{0}\left( t \right)-\gamma\times I_{S_{0}}(t) \right]\times h$ |
| …Test positive (exposed): $T_{E_{0}}$ | $T_{E_{0}}\left( t+h \right)=T_{E_{0}}\left( t \right)-\kappa\times T_{E_{0}}\left( t \right)\times h$ |
| …Test positive (infectious): $T_{I_{0}}$ | $T_{I_{0}}\left( t+h \right)=T_{I_{0}}\left( t \right)-\kappa\times T_{I_{0}}\left( t \right)\times h$ |
| …Isolation housing: $H_{0}$ | $H_{0}\left( t+h \right)=H_{0}\left( t \right)+\left[ \kappa\times\left( T_{E_{0}}\left( t \right)+T_{I_{0}}\left( t \right) \right)+\gamma\times I_{S_{0}}\left( t \right)-\rho_{H}\times H_{0}(t) \right]\times h$ |
| …Quarantine (non-infected): $Q_{s_{0}}$ | $Q_{s_{0}}\left( t+h \right)=Q_{s_{0}}\left( t \right)-\rho_{Q_{S}}\times Q_{s_{0}}\left( t \right)\times h$ |
| …Quarantine (infected): $Q_{E_{0}}$ | $Q_{E_{0}}\left( t+h \right)=Q_{E_{0}}\left( t \right)-\rho_{Q_{E}}\times Q_{E_{0}}\left( t \right)\times h$ |
| …Recovered: $R_{0}$ | $R_{0}\left( t+h \right)=R_{0}\left( t \right)+\left[ \rho_{H}\times H_{0}\left( t \right)+\rho_{Q_{E}}\times Q_{E_{0}}\left( t \right)+\phi\times I_{A_{0}}(t) \right]\times h$ |
| **Fully vaccinated**  **w/out previous infection** |  |
| …Susceptible: $S_{1}$ | $S_{1}\left( t+h \right)=S_{1}\left( t \right)+\left[ \rho_{Q_{S}}\times Q_{s_{1}}\left( t \right)-\beta_{1}\times C\times I_{tot}\times\frac{S_{1}\left( t \right)}{N} \right]\times h$ |
| …Exposed: $E_{1}$ | $E_{1}\left( t+h \right)=E_{1}\left( t \right)+\left[ \beta_{1}\times C\times I_{tot}\times\frac{S_{1}\left( t \right)}{N}-\sigma\times E_{1}(t) \right]\times h$ |
| …Asymptomatic infectious: $I_{A_{1}}$ | $I_{A_{1}}\left( t+h \right)=I_{A_{1}}\left( t \right)+\left[ \left( 1-\alpha\right)\times\sigma\times E_{1}\left( t \right)-\phi\times I_{A_{1}}(t) \right]\times h$ |
| …Symptomatic infectious: $I_{S_{1}}$ | $I_{S_{1}}\left( t+h \right)=I_{S_{1}}\left( t \right)+\left[ \alpha\times\sigma\times E_{1}\left( t \right)-\gamma\times I_{S_{1}}(t) \right]\times h$ |
| …Test positive (exposed): $T_{E_{1}}$ | $T_{E_{1}}\left( t+h \right)=T_{E_{1}}\left( t \right)-\kappa\times T_{E_{1}}\left( t \right)\times h$ |
| …Test positive (infectious): $T_{I_{1}}$ | $T_{I_{1}}\left( t+h \right)=T_{I_{1}}\left( t \right)-\kappa\times T_{I_{1}}\left( t \right)\times h$ |
| …Isolation housing: $H_{1}$ | $H_{1}\left( t+h \right)=H_{1}\left( t \right)+\left[ \kappa\times\left( T_{E_{1}}\left( t \right)+T_{I_{1}}\left( t \right) \right)+\gamma\times I_{S_{1}}\left( t \right)-\rho_{H}\times H_{1}(t) \right]\times h$ |
| …Quarantine (non-infected): $Q_{s_{1}}$ | $Q_{s_{1}}\left( t+h \right)=Q_{s_{1}}\left( t \right)-\rho_{Q_{S}}\times Q_{s_{1}}\left( t \right)\times h$ |
| …Quarantine (infected): $Q_{E_{1}}$ | $Q_{E_{1}}\left( t+h \right)=Q_{E_{1}}\left( t \right)-\rho_{Q_{E}}\times Q_{E_{1}}\left( t \right)\times h$ |
| …Recovered: $R_{1}$ | $R_{1}\left( t+h \right)=R_{1}\left( t \right)+\left[ \rho_{H}\times H_{1}\left( t \right)+\rho_{Q_{E}}\times Q_{E_{1}}\left( t \right)+\phi\times I_{A_{1}}(t) \right]\times h$ |
| **Boosted**  **w/out previous infection** |  |
| …Susceptible: $S_{2}$ | $S_{2}\left( t+h \right)=S_{2}\left( t \right)+\left[ \rho_{Q_{S}}\times Q_{s_{2}}\left( t \right)-\beta_{2}\times C\times I_{tot}\times\frac{S_{2}\left( t \right)}{N} \right]\times h$ |
| …Exposed: $E_{2}$ | $E_{2}\left( t+h \right)=E_{2}\left( t \right)+\left[ \beta_{2}\times C\times I_{tot}\times\frac{S_{2}\left( t \right)}{N}-\sigma\times E_{2}(t) \right]\times h$ |
| …Asymptomatic infectious: $I_{A_{2}}$ | $I_{A_{2}}\left( t+h \right)=I_{A_{2}}\left( t \right)+\left[ \left( 1-\alpha\right)\times\sigma\times E_{2}\left( t \right)-\phi\times I_{A_{2}}(t) \right]\times h$ |
| …Symptomatic infectious: $I_{S_{2}}$ | $I_{S_{2}}\left( t+h \right)=I_{S_{2}}\left( t \right)+\left[ \alpha\times\sigma\times E_{2}\left( t \right)-\gamma\times I_{S_{2}}(t) \right]\times h$ |
| …Test positive (exposed): $T_{E_{2}}$ | $T_{E_{2}}\left( t+h \right)=T_{E_{2}}\left( t \right)-\kappa\times T_{E_{2}}\left( t \right)\times h$ |
| …Test positive (infectious): $T_{I_{2}}$ | $T_{I_{2}}\left( t+h \right)=T_{I_{2}}\left( t \right)-\kappa\times T_{I_{2}}\left( t \right)\times h$ |
| …Isolation housing: $H_{2}$ | $H_{2}\left( t+h \right)=H_{2}\left( t \right)+\left[ \kappa\times\left( T_{E_{2}}\left( t \right)+T_{I_{2}}\left( t \right) \right)+\gamma\times I_{S_{2}}\left( t \right)-\rho_{H}\times H_{2}(t) \right]\times h$ |
| …Quarantine (non-infected): $Q_{s_{2}}$ | $Q_{s_{2}}\left( t+h \right)=Q_{s_{2}}\left( t \right)-\rho_{Q_{S}}\times Q_{s_{2}}\left( t \right)\times h$ |
| …Quarantine (infected): $Q_{E_{2}}$ | $Q_{E_{2}}\left( t+h \right)=Q_{E_{2}}\left( t \right)-\rho_{Q_{E}}\times Q_{E_{2}}\left( t \right)\times h$ |
| …Recovered: $R_{2}$ | $R_{2}\left( t+h \right)=R_{2}\left( t \right)+\left[ \rho_{H}\times H_{2}\left( t \right)+\rho_{Q_{E}}\times Q_{E_{2}}\left( t \right)+\phi\times I_{A_{2}}(t) \right]\times h$ |
| **Previously infected,**  **unvaccinated** |  |
| …Susceptible: $S_{3}$ | $S_{3}\left( t+h \right)=S_{3}\left( t \right)+\left[ \rho_{Q_{S}}\times Q_{s_{3}}\left( t \right)-\beta_{3}\times C\times I_{tot}\times\frac{S_{3}\left( t \right)}{N} \right]\times h$ |
| …Exposed: $E_{3}$ | $E_{3}\left( t+h \right)=E_{3}\left( t \right)+\left[ \beta_{3}\times C\times I_{tot}\times\frac{S_{3}\left( t \right)}{N}-\sigma\times E_{3}(t) \right]\times h$ |
| …Asymptomatic infectious: $I_{A_{3}}$ | $I_{A_{3}}\left( t+h \right)=I_{A_{3}}\left( t \right)+\left[ \left( 1-\alpha\right)\times\sigma\times E_{3}\left( t \right)-\phi\times I_{A_{3}}(t) \right]\times h$ |
| …Symptomatic infectious: $I_{S_{3}}$ | $I_{S_{3}}\left( t+h \right)=I_{S_{3}}\left( t \right)+\left[ \alpha\times\sigma\times E_{3}\left( t \right)-\gamma\times I_{S_{3}}(t) \right]\times h$ |
| …Test positive (exposed): $T_{E_{3}}$ | $T_{E_{3}}\left( t+h \right)=T_{E_{3}}\left( t \right)-\kappa\times T_{E_{3}}\left( t \right)\times h$ |
| …Test positive (infectious): $T_{I_{3}}$ | $T_{I_{3}}\left( t+h \right)=T_{I_{3}}\left( t \right)-\kappa\times T_{I_{3}}\left( t \right)\times h$ |
| …Isolation housing: $H_{3}$ | $H_{3}\left( t+h \right)=H_{3}\left( t \right)+\left[ \kappa\times\left( T_{E_{3}}\left( t \right)+T_{I_{3}}\left( t \right) \right)+\gamma\times I_{S_{3}}\left( t \right)-\rho_{H}\times H_{3}(t) \right]\times h$ |
| …Quarantine (non-infected): $Q_{s_{3}}$ | $Q_{s_{3}}\left( t+h \right)=Q_{s_{3}}\left( t \right)-\rho_{Q_{S}}\times Q_{s_{3}}\left( t \right)\times h$ |
| …Quarantine (infected): $Q_{E_{3}}$ | $Q_{E_{3}}\left( t+h \right)=Q_{E_{3}}\left( t \right)-\rho_{Q_{E}}\times Q_{E_{3}}\left( t \right)\times h$ |
| …Recovered: $R_{3}$ | $R_{3}\left( t+h \right)=R_{3}\left( t \right)+\left[ \rho_{H}\times H_{3}\left( t \right)+\rho_{Q_{E}}\times Q_{E_{3}}\left( t \right)+\phi\times I_{A_{3}}(t) \right]\times h$ |
| **Fully vaccinated**  **w/ previous infection** |  |
| …Susceptible: $S_{4}$ | $S_{4}\left( t+h \right)=S_{4}\left( t \right)+\left[ \rho_{Q_{S}}\times Q_{s_{4}}\left( t \right)-\beta_{4}\times C\times I_{tot}\times\frac{S_{4}\left( t \right)}{N} \right]\times h$ |
| …Exposed: $E_{4}$ | $E_{4}\left( t+h \right)=E_{4}\left( t \right)+\left[ \beta_{4}\times C\times I_{tot}\times\frac{S_{4}\left( t \right)}{N}-\sigma\times E_{4}(t) \right]\times h$ |
| …Asymptomatic infectious: $I_{A_{4}}$ | $I_{A_{4}}\left( t+h \right)=I_{A_{4}}\left( t \right)+\left[ \left( 1-\alpha\right)\times\sigma\times E_{4}\left( t \right)-\phi\times I_{A_{4}}(t) \right]\times h$ |
| …Symptomatic infectious: $I_{S_{4}}$ | $I_{S_{4}}\left( t+h \right)=I_{S_{4}}\left( t \right)+\left[ \alpha\times\sigma\times E_{4}\left( t \right)-\gamma\times I_{S_{4}}(t) \right]\times h$ |
| …Test positive (exposed): $T_{E_{4}}$ | $T_{E_{4}}\left( t+h \right)=T_{E_{4}}\left( t \right)-\kappa\times T_{E_{4}}\left( t \right)\times h$ |
| …Test positive (infectious): $T_{I_{4}}$ | $T_{I_{4}}\left( t+h \right)=T_{I_{4}}\left( t \right)-\kappa\times T_{I_{4}}\left( t \right)\times h$ |
| …Isolation housing: $H_{4}$ | $H_{4}\left( t+h \right)=H_{4}\left( t \right)+\left[ \kappa\times\left( T_{E_{4}}\left( t \right)+T_{I_{4}}\left( t \right) \right)+\gamma\times I_{S_{4}}\left( t \right)-\rho_{H}\times H_{4}(t) \right]\times h$ |
| …Quarantine (non-infected): $Q_{s_{4}}$ | $Q_{s_{4}}\left( t+h \right)=Q_{s_{4}}\left( t \right)-\rho_{Q_{S}}\times Q_{s_{4}}\left( t \right)\times h$ |
| …Quarantine (infected): $Q_{E_{4}}$ | $Q_{E_{4}}\left( t+h \right)=Q_{E_{4}}\left( t \right)-\rho_{Q_{E}}\times Q_{E_{4}}\left( t \right)\times h$ |
| …Recovered: $R_{4}$ | $R_{4}\left( t+h \right)=R_{4}\left( t \right)+\left[ \rho_{H}\times H_{4}\left( t \right)+\rho_{Q_{E}}\times Q_{E_{4}}\left( t \right)+\phi\times I_{A_{4}}(t) \right]\times h$ |
| **Boosted**  **w/ previous infection** |  |
| …Susceptible: $S_{5}$ | $S_{5}\left( t+h \right)=S_{5}\left( t \right)+\left[ \rho_{Q_{S}}\times Q_{s_{5}}\left( t \right)-\beta_{5}\times C\times I_{tot}\times\frac{S_{5}\left( t \right)}{N} \right]\times h$ |
| …Exposed: $E_{5}$ | $E_{5}\left( t+h \right)=E_{5}\left( t \right)+\left[ \beta_{5}\times C\times I_{tot}\times\frac{S_{5}\left( t \right)}{N}-\sigma\times E_{5}(t) \right]\times h$ |
| …Asymptomatic infectious: $I_{A_{5}}$ | $I_{A_{5}}\left( t+h \right)=I_{A_{5}}\left( t \right)+\left[ \left( 1-\alpha\right)\times\sigma\times E_{5}\left( t \right)-\phi\times I_{A_{5}}(t) \right]\times h$ |
| …Symptomatic infectious: $I_{S_{5}}$ | $I_{S_{5}}\left( t+h \right)=I_{S_{5}}\left( t \right)+\left[ \alpha\times\sigma\times E_{5}\left( t \right)-\gamma\times I_{S_{5}}(t) \right]\times h$ |
| …Test positive (exposed): $T_{E_{5}}$ | $T_{E_{5}}\left( t+h \right)=T_{E_{5}}\left( t \right)-\kappa\times T_{E_{5}}\left( t \right)\times h$ |
| …Test positive (infectious): $T_{I_{5}}$ | $T_{I_{5}}\left( t+h \right)=T_{I_{5}}\left( t \right)-\kappa\times T_{I_{5}}\left( t \right)\times h$ |
| …Isolation housing: $H_{5}$ | $H_{5}\left( t+h \right)=H_{5}\left( t \right)+\left[ \kappa\times\left( T_{E_{5}}\left( t \right)+T_{I_{5}}\left( t \right) \right)+\gamma\times I_{S_{5}}\left( t \right)-\rho_{H}\times H_{5}(t) \right]\times h$ |
| …Quarantine (non-infected): $Q_{s_{5}}$ | $Q_{s_{5}}\left( t+h \right)=Q_{s_{5}}\left( t \right)-\rho_{Q_{S}}\times Q_{s_{5}}\left( t \right)\times h$ |
| …Quarantine (infected): $Q_{E_{5}}$ | $Q_{E_{5}}\left( t+h \right)=Q_{E_{5}}\left( t \right)-\rho_{Q_{E}}\times Q_{E_{5}}\left( t \right)\times h$ |
| …Recovered: $R_{5}$ | $R_{5}\left( t+h \right)=R_{5}\left( t \right)+\left[ \rho_{H}\times H_{5}\left( t \right)+\rho_{Q_{E}}\times Q_{E_{5}}\left( t \right)+\phi\times I_{A_{5}}(t) \right]\times h$ |
| **If** $\boldsymbol{mod}\left( \boldsymbol{t,1} \right)\boldsymbol{=0}$**, then for** $\boldsymbol{j=0, 1,\ldots, 5,}$ | |
| Exposed: | $E_{j}\left( t \right)=E_{j}\left( t \right)-E_{j}\left( t \right)\times p\times se_{E}$ |
| Asymptomatic infectious: | $I_{A_{j}}\left( t \right)=I_{A_{j}}\left( t \right)-I_{A_{j}}\left( t \right)\times p\times se_{I}$ |
| Symptomatic infectious: | $I_{S_{j}}\left( t \right)=I_{S_{j}}\left( t \right)-I_{S_{j}}\left( t \right)\times p\times se_{I}$ |
| Test positive (exposed): | $T_{E_{j}}\left( t \right)=T_{E_{j}}\left( t \right)+E_{j}\left( t \right)\times p\times se_{E}$ |
| Test positive (infectious): | $T_{I_{j}}\left( t \right)=T_{I_{j}}\left( t \right)+\left[ I_{A_{j}}\left( t \right)+I_{S_{j}}\left( t \right) \right]\times p\times se_{I}$ |

* $I_{tot}\left( t \right)=\sum_{j=0}^{5} \left[ I_{A_{j}}\left( t \right)+I_{S_{j}}\left( t \right)+T_{I_{j}}\left( t \right) \right]$ represents the total number of infectious individuals at time step *t*. And $N=\sum_{j=0}^{5} N_{j}$ is the vector of sub-population sizes.

**Table S2-S5.**

**Initial values in the compartment model.**

**Table S2. Initial values in the compartment model in Clemson University, Spring ’22 analysis.**

|  | **In-state residential** | **Out-of-state residential** | **Non-residential** | **Faculty** | **Staff** | **Community** |
| --- | --- | --- | --- | --- | --- | --- |
| **Susceptible** |  |  |  |  |  |  |
| S_0_ | 671 | 150 | 2246 | 133 | 507 | 561 |
| S_1_ | 989 | 391 | 2417 | 827 | 1142 | 1586 |
| S_2_ | 90 | 37 | 206 | 191 | 275 | 362 |
| S_3_ | 187 | 60 | 1206 | 11 | 194 | 167 |
| S_4_ | 153 | 111 | 865 | 59 | 172 | 182 |
| S_5_ | 14 | 10 | 75 | 14 | 42 | 41 |
| **Exposed** |  |  |  |  |  |  |
| E_0_ | 55 | 15 | 176 | 3 | 9 | 145 |
| E_1_ | 82 | 42 | 185 | 12 | 21 | 409 |
| E_2_ | 6 | 3 | 15 | 3 | 6 | 94 |
| E_3_ | 15 | 6 | 94 | 0 | 3 | 42 |
| E_4_ | 12 | 9 | 64 | 0 | 3 | 48 |
| E_5_ | 0 | 0 | 6 | 0 | 0 | 12 |
| **Symptomatic** |  |  |  |  |  |  |
| $I_{S_{0}}$ | 12 | 4 | 40 | 1 | 2 | 33 |
| $I_{S_{1}}$ | 19 | 9 | 42 | 3 | 4 | 93 |
| $I_{S_{2}}$ | 1 | 1 | 3 | 1 | 1 | 22 |
| $I_{S_{3}}$ | 3 | 1 | 21 | 0 | 1 | 9 |
| $I_{S_{4}}$ | 3 | 2 | 14 | 0 | 1 | 10 |
| $I_{S_{5}}$ | 0 | 0 | 1 | 0 | 0 | 2 |
| **Asymptomatic** |  |  |  |  |  |  |
| $I_{A_{0}}$ | 62 | 19 | 200 | 3 | 11 | 165 |
| $I_{A_{1}}$ | 92 | 48 | 210 | 15 | 23 | 465 |
| $I_{A_{2}}$ | 7 | 4 | 16 | 3 | 5 | 106 |
| $I_{A_{3}}$ | 16 | 6 | 105 | 0 | 4 | 48 |
| $I_{A_{4}}$ | 13 | 11 | 73 | 1 | 3 | 54 |
| $I_{A_{5}}$ | 1 | 1 | 6 | 0 | 1 | 12 |
| **Recovered** |  |  |  |  |  |  |
| R_0_ | 636 | 222 | 2044 | 35 | 191 | 2518 |
| R_1_ | 924 | 575 | 2126 | 196 | 343 | 7117 |
| R_2_ | 73 | 43 | 168 | 43 | 74 | 1626 |
| R_3_ | 158 | 72 | 1052 | 4 | 52 | 748 |
| R_4_ | 136 | 137 | 742 | 14 | 47 | 817 |
| R_5_ | 11 | 11 | 59 | 3 | 9 | 187 |

**Table S3. Initial values in the compartment model in University of Georgia, Spring ’22 analysis.**

|  | **In-state residential** | **Out-of-state residential** | **Non-residential** | **Faculty** | **Staff** | **Community** |
| --- | --- | --- | --- | --- | --- | --- |
| **Susceptible** |  |  |  |  |  |  |
| S_0_ | 707 | 137 | 2745 | 271 | 1060 | 22565 |
| S_1_ | 669 | 220 | 2232 | 1133 | 1499 | 26437 |
| S_2_ | 1291 | 596 | 3193 | 1180 | 1725 | 24697 |
| S_3_ | 206 | 65 | 1478 | 21 | 401 | 9707 |
| S_4_ | 136 | 91 | 881 | 84 | 222 | 6245 |
| S_5_ | 191 | 147 | 1135 | 84 | 249 | 5835 |
| **Exposed** |  |  |  |  |  |  |
| E_0_ | 103 | 36 | 251 | 6 | 17 | 60 |
| E_1_ | 180 | 92 | 244 | 14 | 30 | 70 |
| E_2_ | 9 | 7 | 13 | 2 | 1 | 66 |
| E_3_ | 26 | 10 | 135 | 1 | 6 | 26 |
| E_4_ | 16 | 15 | 61 | 0 | 6 | 17 |
| E_5_ | 1 | 0 | 1 | 0 | 2 | 16 |
| **Symptomatic** |  |  |  |  |  |  |
| $I_{S_{0}}$ | 68 | 24 | 168 | 4 | 11 | 40 |
| $I_{S_{1}}$ | 120 | 61 | 163 | 9 | 20 | 47 |
| $I_{S_{2}}$ | 6 | 5 | 9 | 1 | 1 | 44 |
| $I_{S_{3}}$ | 17 | 7 | 90 | 1 | 4 | 17 |
| $I_{S_{4}}$ | 10 | 10 | 41 | 0 | 4 | 11 |
| $I_{S_{5}}$ | 1 | 0 | 1 | 0 | 1 | 10 |
| **Asymptomatic** |  |  |  |  |  |  |
| $I_{A_{0}}$ | 342 | 120 | 838 | 19 | 57 | 201 |
| $I_{A_{1}}$ | 601 | 305 | 814 | 46 | 100 | 235 |
| $I_{A_{2}}$ | 31 | 25 | 43 | 5 | 3 | 220 |
| $I_{A_{3}}$ | 86 | 34 | 450 | 3 | 19 | 86 |
| $I_{A_{4}}$ | 52 | 49 | 203 | 0 | 19 | 56 |
| $I_{A_{5}}$ | 3 | 0 | 3 | 0 | 5 | 52 |
| **Recovered** |  |  |  |  |  |  |
| R_0_ | 864 | 287 | 2817 | 77 | 425 | 7220 |
| R_1_ | 909 | 584 | 2350 | 321 | 581 | 8460 |
| R_2_ | 1178 | 683 | 2475 | 249 | 408 | 7903 |
| R_3_ | 215 | 97 | 1436 | 8 | 116 | 3107 |
| R_4_ | 157 | 151 | 869 | 23 | 76 | 1998 |
| R_5_ | 174 | 167 | 875 | 16 | 54 | 1867 |

**Table S4. Initial values in the compartment model in Pennsylvania State University, Spring ’22 analysis.**

|  | **In-state residential** | **Out-of-state residential** | **Non-residential** | **Faculty** | **Staff** | **Community** |
| --- | --- | --- | --- | --- | --- | --- |
| **Susceptible** |  |  |  |  |  |  |
| S_0_ | 292 | 22 | 1120 | 185 | 672 | 3444 |
| S_1_ | 1882 | 809 | 4727 | 1670 | 2281 | 11147 |
| S_2_ | 2305 | 1152 | 5332 | 1680 | 2454 | 10415 |
| S_3_ | 101 | 28 | 638 | 13 | 275 | 1453 |
| S_4_ | 304 | 233 | 1728 | 119 | 340 | 2668 |
| S_5_ | 341 | 277 | 1882 | 119 | 357 | 2492 |
| **Exposed** |  |  |  |  |  |  |
| E_0_ | 51 | 18 | 126 | 4 | 10 | 9 |
| E_1_ | 90 | 46 | 122 | 8 | 18 | 30 |
| E_2_ | 5 | 4 | 6 | 1 | 0 | 28 |
| E_3_ | 13 | 5 | 67 | 0 | 4 | 4 |
| E_4_ | 8 | 7 | 31 | 0 | 4 | 7 |
| E_5_ | 0 | 0 | 0 | 0 | 1 | 7 |
| **Symptomatic** |  |  |  |  |  |  |
| $I_{S_{0}}$ | 34 | 12 | 84 | 2 | 7 | 6 |
| $I_{S_{1}}$ | 60 | 31 | 81 | 6 | 12 | 20 |
| $I_{S_{2}}$ | 3 | 2 | 4 | 1 | 0 | 19 |
| $I_{S_{3}}$ | 9 | 3 | 45 | 0 | 2 | 3 |
| $I_{S_{4}}$ | 5 | 5 | 20 | 0 | 2 | 5 |
| $I_{S_{5}}$ | 0 | 0 | 0 | 0 | 1 | 4 |
| **Asymptomatic** |  |  |  |  |  |  |
| $I_{A_{0}}$ | 171 | 60 | 419 | 12 | 35 | 31 |
| $I_{A_{1}}$ | 300 | 153 | 407 | 28 | 62 | 99 |
| $I_{A_{2}}$ | 15 | 12 | 22 | 3 | 2 | 93 |
| $I_{A_{3}}$ | 43 | 17 | 225 | 2 | 12 | 13 |
| $I_{A_{4}}$ | 26 | 25 | 102 | 0 | 12 | 24 |
| $I_{A_{5}}$ | 2 | 0 | 2 | 0 | 3 | 22 |
| **Recovered** |  |  |  |  |  |  |
| R_0_ | 461 | 171 | 1568 | 65 | 390 | 1102 |
| R_1_ | 1626 | 1000 | 3836 | 443 | 796 | 3568 |
| R_2_ | 1569 | 873 | 3517 | 342 | 558 | 3332 |
| R_3_ | 101 | 48 | 772 | 9 | 95 | 464 |
| R_4_ | 246 | 231 | 1356 | 31 | 106 | 853 |
| R_5_ | 230 | 209 | 1236 | 23 | 76 | 798 |

**Table S5. Initial values in the compartment model in Clemson University, Fall ’22 analysis.**

|  | **In-state residential** | **Out-of-state residential** | **Non-residential** | **Faculty** | **Staff** | **Community** |
| --- | --- | --- | --- | --- | --- | --- |
| **Susceptible** |  |  |  |  |  |  |
| S_0_ | 609 | 218 | 1400 | 71 | 198 | 382 |
| S_1_ | 625 | 286 | 1258 | 232 | 271 | 454 |
| S_2_ | 619 | 283 | 1348 | 282 | 347 | 424 |
| S_3_ | 628 | 259 | 1766 | 26 | 215 | 399 |
| S_4_ | 481 | 335 | 1244 | 78 | 194 | 338 |
| S_5_ | 451 | 322 | 1240 | 87 | 205 | 316 |
| **Exposed** |  |  |  |  |  |  |
| E_0_ | 9 | 0 | 82 | 9 | 36 | 124 |
| E_1_ | 18 | 9 | 67 | 36 | 48 | 148 |
| E_2_ | 0 | 0 | 18 | 6 | 12 | 136 |
| E_3_ | 0 | 0 | 18 | 0 | 12 | 130 |
| E_4_ | 0 | 6 | 36 | 9 | 12 | 109 |
| E_5_ | 0 | 0 | 0 | 0 | 0 | 103 |
| **Symptomatic** |  |  |  |  |  |  |
| $I_{S_{0}}$ | 2 | 0 | 19 | 2 | 8 | 28 |
| $I_{S_{1}}$ | 4 | 2 | 15 | 8 | 11 | 33 |
| $I_{S_{2}}$ | 0 | 0 | 4 | 1 | 3 | 31 |
| $I_{S_{3}}$ | 0 | 0 | 4 | 0 | 3 | 30 |
| $I_{S_{4}}$ | 0 | 1 | 8 | 2 | 3 | 25 |
| $I_{S_{5}}$ | 0 | 0 | 0 | 0 | 0 | 24 |
| **Asymptomatic** |  |  |  |  |  |  |
| $I_{A_{0}}$ | 10 | 0 | 93 | 10 | 41 | 140 |
| $I_{A_{1}}$ | 21 | 10 | 77 | 41 | 57 | 167 |
| $I_{A_{2}}$ | 0 | 0 | 21 | 5 | 15 | 156 |
| $I_{A_{3}}$ | 0 | 0 | 21 | 0 | 15 | 147 |
| $I_{A_{4}}$ | 0 | 5 | 41 | 10 | 15 | 125 |
| $I_{A_{5}}$ | 0 | 0 | 0 | 0 | 0 | 116 |
| **Recovered** |  |  |  |  |  |  |
| R_0_ | 209 | 76 | 561 | 75 | 247 | 2241 |
| R_1_ | 227 | 99 | 602 | 290 | 416 | 2666 |
| R_2_ | 218 | 97 | 507 | 280 | 376 | 2492 |
| R_3_ | 217 | 88 | 667 | 25 | 228 | 2352 |
| R_4_ | 166 | 115 | 482 | 78 | 212 | 1987 |
| R_5_ | 154 | 110 | 459 | 80 | 206 | 1856 |

**Table S6-S8.**

**Model input parameters and references.**

**Table S6. Input parameters in Clemson University, Spring ’22 analysis.**

| **Model parameter** | **Input** |
| --- | --- |
| Disease dynamics |  |
| …Mean incubation time (days): $1/\sigma$ | 3 days ^5^ |
| …Mean asymptomatic infectious time (days): $1/\phi$ | 10 days ^6^ |
| …Mean symptomatic infection time before detection/isolation (days): $1/\gamma$ | 3 days ^7^ |
| …Lag between test and results: $1/\kappa$ | 1 day ^¶^ |
| …Days in isolation housing: ${1/\rho}_{H}$ | 5 days ^¶^ |
| …Days in quarantine for non-infected individuals: $1/\rho_{Q_{S}}$ | 5 days ^¶^ |
| …Days in quarantine for infected individuals: $1/\rho_{Q_{E}}$ | 7.5 days ^¶^ |
| …Proportion of infections detected through voluntary testing: $\alpha$ ^¶^ |  |
| ……Residential students | 0.10 |
| ……Non-residential students | 0.10 |
| ……Faculty | 0.15 |
| ……Staff | 0.15 |
| ……Community | 0.15 |
| Disease reproductive number: $R_{0}$^*^ |  |
| …Residential students | 10.0 |
| …Non-residential students | 8.3 |
| …Faculty | 4.1 |
| …Staff | 5.4 |
| …Community | 5.4 |
| Transmission rate ^**^ |  |
| …Unprotected: $\beta_{0}$ | $R_{0}\cdot\phi$ ^8^ |
| …Fully vaccinated w/out previous infection: $\beta_{1}$ | $\beta_{0}\cdot(1-hr_{1})$ |
| …Boosted w/out previous infection: $\beta_{2}$ | $\beta_{0}\cdot(1-{hr}_{2})$ |
| …Previously infected, unvaccinated: $\beta_{3}$ | $\beta_{0}\cdot(1-{hr}_{3})$ |
| …Fully vaccinated w/ previous infection: $\beta_{4}$ | $\beta_{0}\cdot(1-{hr}_{4})$ |
| …Boosted w/ previous infection: $\beta_{5}$ | $\beta_{0}\cdot(1-{hr}_{5})$ |
| Contact matrix: $C$ ^†^ | Varies |
| Daily random tests: $p$ ^‡^ |  |
| …Residential students | 14.3% |
| …Non-residential students | 14.3% |
| …Faculty | 14.3% |
| …Staff | 14.3% |
| …Community | 0.1% |
| Test sensitivity |  |
| …Exposed (s$e_{E}$) | 33% ^9^ |
| …Infectious (s$e_{I}$) | 95% ^10^ |

¶ Based on empirical data at Clemson University.

* Validated internally using Fall 2021 data at Clemson University.

** The parameters ${hr}_{j}$ for $j=0, 1,\ldots, 5,$ represent the percent reduction in the infection rate relative to unprotected, estimated using Cox proportional hazards model based on Clemson University data between December 31, 2021 and January 9, 2022 (Supplementary Appendix).

† See Supplementary Appendix for specification of contact matrices.

‡ Daily proportion tested for individuals affiliated with Clemson University is set to $\frac{1}{7}\approx14.3\%$ to represent the weekly testing strategy.

**Table S7. Input parameters in UGA and PSU, Spring 2022 analyses.**

| **Model parameter** | **Input** |
| --- | --- |
| Disease dynamics |  |
| …Mean incubation time (days): $1/\sigma$ | 3 days ^5^ |
| …Mean asymptomatic infectious time (days): $1/\phi$ | 10 days ^6^ |
| …Mean symptomatic infection time before detection/isolation (days): $1/\gamma$ | 3 days ^7^ |
| …Lag between test and results: $1/\kappa$ | 1 day ^¶^ |
| …Days in isolation housing: ${1/\rho}_{H}$ | 5 days ^¶^ |
| …Days in quarantine for non-infected individuals: $1/\rho_{Q_{S}}$ | 5 days ^¶^ |
| …Days in quarantine for infected individuals: $1/\rho_{Q_{E}}$ | 7.5 days ^¶^ |
| …Proportion of infections detected through voluntary testing: $\alpha$ ^¶^ |  |
| ……Residential students | 0.10 |
| ……Non-residential students | 0.10 |
| ……Faculty | 0.15 |
| ……Staff | 0.15 |
| ……Community | 0.15 |
| Disease reproductive number: $R_{0}$^*^ |  |
| …Residential students | 10.0 |
| …Non-residential students | 8.3 |
| …Faculty | 4.1 |
| …Staff | 5.4 |
| …Community | 5.4 |
| Transmission rate ^**^ |  |
| …Unprotected: $\beta_{0}$ | $R_{0}\cdot\phi$ ^8^ |
| …Fully vaccinated w/out previous infection: $\beta_{1}$ | $\beta_{0}\cdot(1-hr_{1})$ |
| …Boosted w/out previous infection: $\beta_{2}$ | $\beta_{0}\cdot(1-{hr}_{2})$ |
| …Previously infected, unvaccinated: $\beta_{3}$ | $\beta_{0}\cdot(1-{hr}_{3})$ |
| …Fully vaccinated w/ previous infection: $\beta_{4}$ | $\beta_{0}\cdot(1-{hr}_{4})$ |
| …Boosted w/ previous infection: $\beta_{5}$ | $\beta_{0}\cdot(1-{hr}_{5})$ |
| Contact matrix: $C$ ^†^ | Varies |
| Daily random surveillance tests: $p$ ^‡^ |  |
| …Residential students | 0.1% |
| …Non-residential students | 0.1% |
| …Faculty | 0.1% |
| …Staff | 0.1% |
| …Community | 0.1% |
| Test sensitivity |  |
| …Exposed (s$e_{E}$) | 33% ^9^ |
| …Infectious (s$e_{I}$) | 95% ^10^ |

¶ Based on empirical data at Clemson University.

* Validated using Fall 2021 data at Clemson University.

** The parameters ${hr}_{j}$ for $j=0, 1,\ldots, 5,$ represent the percent reduction in the infection rate relative to unprotected, estimated using Cox proportional hazards model based on Clemson University data between December 31, 2021 and January 9, 2022 (Supplementary Appendix).

† See Supplementary Appendix for specification of contact matrices.

‡ Daily proportion tested for individuals is set to 0.1% to reflect voluntary testing.

**Table S8. Input parameters in Clemson University, Fall 2022 analysis.**

| **Model parameter** | **Input** |
| --- | --- |
| Disease dynamics |  |
| …Mean incubation time (days): $1/\sigma$ | 3 days ^5^ |
| …Mean asymptomatic infectious time (days): $1/\phi$ | 10 days ^6^ |
| …Mean symptomatic infection time before detection/isolation (days): $1/\gamma$ | 3 days ^7^ |
| …Lag between test and results: $1/\kappa$ | 1 day ^¶^ |
| …Days in isolation housing: ${1/\rho}_{H}$ | 5 days ^¶^ |
| …Days in quarantine for non-infected individuals: $1/\rho_{Q_{S}}$ | 5 days ^¶^ |
| …Days in quarantine for infected individuals: $1/\rho_{Q_{E}}$ | 7.5 days ^¶^ |
| …Proportion of infections detected through voluntary testing: $\alpha$ ^¶^ |  |
| ……Residential students | 0.10 |
| ……Non-residential students | 0.10 |
| ……Faculty | 0.15 |
| ……Staff | 0.15 |
| ……Community | 0.15 |
| Disease reproductive number: $R_{0}$^*^ |  |
| …Residential students | 10.0 |
| …Non-residential students | 8.3 |
| …Faculty | 4.1 |
| …Staff | 5.4 |
| …Community | 5.4 |
| Transmission rate |  |
| …Unprotected: $\beta_{0}$ | $R_{0}\cdot\phi$ ^8^ |
| …Fully vaccinated w/out previous infection: $\beta_{1}$ | $0.35\times\beta_{0}$ ^11,12^ |
| …Boosted w/out previous infection: $\beta_{2}$ | $0.46\times\beta_{0}$ ^11,12^ |
| …Previously infected, unvaccinated: $\beta_{3}$ | $0.72\times\beta_{0}$ ^11,12^ |
| …Fully vaccinated w/ previous infection: $\beta_{4}$ | $0.82\times\beta_{0}$ ^11,12^ |
| …Boosted w/ previous infection: $\beta_{5}$ | $0.84\times\beta_{0}$ ^11,12^ |
| Contact matrix: $C$ ^†^ | Varies |
| Daily random tests: $p$ ^‡^ |  |
| …Residential students | 0.1% |
| …Non-residential students | 0.1% |
| …Faculty | 0.1% |
| …Staff | 0.1% |
| …Community | 0.1% |
| Test sensitivity |  |
| …Exposed (s$e_{E}$) | 33% ^9^ |
| …Infectious (s$e_{I}$) | 95% ^10^ |

¶ Based on empirical data at Clemson University.

* Validated using Fall 2021 data at Clemson University.

† See Supplementary Appendix for specification of contact matrices.

‡ Daily proportion tested for individuals is set to 0.1% to reflect voluntary testing.

**Table S9-S12. Estimated individuals in each protection level.** For $j=0,1,2,\ldots,5$, *N_j_* represents the number of individuals that are unprotected, fully vaccinated without previous infections, boosted without previous infections, previously infected only, fully vaccinated with previous infections, and boosted with previous infections, respectively.

**Table S9. Estimated individuals in each protection level in Clemson University, Spring 2022 analysis.**

|  | **In-state residential** | **Out-of-state residential** | **Non-residential** | **Faculty** | **Staff** | **Community** |
| --- | --- | --- | --- | --- | --- | --- |
| N_0_ | 1557 | 466 | 5077 | 182 | 763 | 3422 |
| N_1_ | 2329 | 1224 | 5333 | 1074 | 1595 | 9670 |
| N_2_ | 186 | 98 | 426 | 246 | 365 | 2210 |
| N_3_ | 410 | 162 | 2670 | 17 | 263 | 1014 |
| N_4_ | 343 | 291 | 1862 | 75 | 232 | 1111 |
| N_5_ | 27 | 23 | 149 | 17 | 53 | 254 |
| ***Total*** | ***4852*** | ***2264*** | ***15517*** | ***1611*** | ***3271*** | ***17681*** |

**Table S10. Estimated individuals in each protection level in University of Georgia, Spring 2022 analysis.**

|  | **In-state residential** | **Out-of-state residential** | **Non-residential** | **Faculty** | **Staff** | **Community** |
| --- | --- | --- | --- | --- | --- | --- |
| N_0_ | 2205 | 660 | 7188 | 384 | 1609 | 30086 |
| N_1_ | 2701 | 1420 | 6156 | 1544 | 2292 | 35249 |
| N_2_ | 2524 | 1326 | 5751 | 1442 | 2142 | 32930 |
| N_3_ | 581 | 230 | 3780 | 36 | 555 | 12943 |
| N_4_ | 397 | 337 | 2159 | 108 | 333 | 8327 |
| N_5_ | 371 | 315 | 2017 | 100 | 312 | 7780 |
| ***Total*** | ***8779*** | ***4288*** | ***27051*** | ***3614*** | ***7243*** | ***127315*** |

**Table S11. Estimated individuals in each protection level in Pennsylvania State University, Spring 2022 analysis.**

|  | **In-state residential** | **Out-of-state residential** | **Non-residential** | **Faculty** | **Staff** | **Community** |
| --- | --- | --- | --- | --- | --- | --- |
| N_0_ | 565 | 169 | 1843 | 275 | 1153 | 2794 |
| N_1_ | 4578 | 2406 | 10433 | 2176 | 3231 | 15922 |
| N_2_ | 4277 | 2248 | 9747 | 2032 | 3018 | 14874 |
| N_3_ | 149 | 59 | 969 | 26 | 397 | 1116 |
| N_4_ | 673 | 571 | 3660 | 151 | 470 | 3853 |
| N_5_ | 629 | 533 | 3419 | 142 | 439 | 3601 |
| ***Total*** | ***10871*** | ***5986*** | ***30071*** | ***4802*** | ***8708*** | ***42160*** |

**Table S12. Estimated individuals in each protection level in Clemson University, Fall 2022 analysis.**

|  | **In-state residential** | **Out-of-state residential** | **Non-residential** | **Faculty** | **Staff** | **Community** |
| --- | --- | --- | --- | --- | --- | --- |
| N_0_ | 840 | 294 | 2161 | 169 | 537 | 2915 |
| N_1_ | 896 | 407 | 2034 | 614 | 808 | 3468 |
| N_2_ | 837 | 380 | 1900 | 574 | 755 | 3239 |
| N_3_ | 845 | 347 | 2477 | 51 | 478 | 3060 |
| N_4_ | 647 | 463 | 1818 | 179 | 440 | 2584 |
| N_5_ | 605 | 432 | 1699 | 167 | 411 | 2415 |
| ***Total*** | ***4670*** | ***2323*** | ***12089*** | ***1754*** | ***3429*** | ***17681*** |

**Table S13-S15.**

**Results of sensitivity analyses.**

**Table S13. Results of sensitivity analyses in Clemson University, Spring ’22 analysis.**

|  | **Student cases** | **Employee cases** | **Student Maximum Isolation/Quarantine** |
| --- | --- | --- | --- |
| Observed | 4876 | 876 | 1881 |
| Predicted | 4947 | 891 | 1710 |
| **Sensitivity analyses^*^** |  |  |  |
| No protection from previous infection | 5227 (7.2%) | 926 (5.7%) | 1710 (0.0%) |
| No adjustment to recovered individuals | 11312 (132.0%) | 3885 (343.0%) | 2391 (39.8%) |
| No nonpharmaceutical intervention | 7476 (53.3%) | 1869 (113.4%) | 1966 (15.0%) |

* Numbers in parentheses for student and employee cases are percent-increments relative to observed numbers. Numbers in parentheses for student maximum isolation/quarantine are percent-increments relative to predicted cases.

**Table S14. Results of sensitivity analyses in University of Georgia and Pennsylvania State University, Spring ’22 analyses.**

|  | **UGA** | **PSU** |
| --- | --- | --- |
| Observed | 2550 | 1708 |
| Predicted | 2492 | 1983 |
| **Sensitivity analyses^*^** |  |  |
| No protection from previous infection | 2801 (9.8%) | 2375 (39.1%) |
| No adjustment to recovered individuals | 4187 (64.2%) | 3657 (114.1%) |

* Numbers in parentheses are percent-increments relative to observed numbers.

**Table S15. Results of sensitivity analyses in Clemson University, Fall ’22 analysis.**

|  | **Student cases** | **Employee cases** | **Student Maximum Isolation/Quarantine** |
| --- | --- | --- | --- |
| Observed | 634 | 118 | 249 |
| Predicted | 622 | 183 | 199 |
| **Sensitivity analyses^*^** |  |  |  |
| No protection from previous infection | 994 (56.8%) | 192 (62.7%) | 473 (137.7%) |
| No adjustment to recovered individuals | 1253 (97.6%) | 521 (341.5%) | 391 (96.5%) |
| With nonpharmaceutical intervention | 331 (-47.8%) | 71 (-39.8%) | 155 (-22.1%) |

* Numbers in parentheses for student and employee cases are percent-increments relative to observed numbers. Numbers in parentheses for student maximum isolation/quarantine are percent-increments relative to predicted cases.

**Table S16. List of input parameters and initial states that can be modified by the users in the toolkit.**

| **Parameters** | **Values** |
| --- | --- |
| Testing strategy | Weekly/Bi-weekly/Monthly/Voluntary |
| Percent students recovered within last 90 days | 0-100 |
| Percent employees recovered within last 90 days | 0-100 |
| Proportion of infections detected through voluntary testing (by subpopulation) | 0.05-0.8 |
| Add additional recovered | Yes/No |
| Proportion of additional recovered | 0-1 |
| Basic reproduction number (R_0_) | 0.5-15 |
| Community baseline infection rate | 0-0.25 |
| Community baseline recovery rate | 0-0.5 |
| Time between arrival of students (baseline) and onset of surveillance testing (days) | 0-14 |
| Number of contacts per person | 0-10 |
| Time in quarantine for non-infected individuals | 1-14 |
| Time in quarantine for infected individuals | 7-15 |
| Days in isolation | 3-14 |
| Test turnaround time | 0.5-3 |
| Test sensitivity (infectious) | 0.5-0.99 |
| Test sensitivity (exposed) | 0.1-0.99 |
| Exposure period | 2-7 |
| Infectious period for aymptomatic cases | 5-14 |
| Lag between infection and detection for symptomatic cases | 1-14 |
| Prediction length (weeks) | 2-20 |
|  |  |
| **Initial states** |  |
| Number of students/employees­^*^ | |
| Number of residential students/non-residential students/faculty/staff^**^ | |
| Community population size | |
| Percent fully vaccinated among students/employees | |
| Percent boosted among students/employees | |
| Currently infected students/employees | |
| Currently isolated students/employees | |
| Currently quarantined students/employees | |

* If choosing “Student/Employee” in “Input by affiliation”

** If choosing “Residential /Non-Residential /Faculty/Staff”

**REFERENCES**

1. Lloyd, A. L. & Jansen, V. A. A. Spatiotemporal dynamics of epidemics: synchrony in metapopulation models. *Math. Biosci.* **188**, 1–16 (2004).

2. Census Bureau Search. https://data.census.gov/cedsci/all?g=1600000US4514950.

3. Pearson, C. A. B. *et al.* Bounding the levels of transmissibility &amp; immune evasion of the Omicron variant in South Africa. 2021.12.19.21268038 Preprint at https://doi.org/10.1101/2021.12.19.21268038 (2021).

4. Ito, K., Piantham, C. & Nishiura, H. Estimating relative generation times and reproduction numbers of Omicron BA.1 and BA.2 with respect to Delta variant in Denmark. *Math. Biosci. Eng. MBE* **19**, 9005–9017 (2022).

5. Paltiel, A. D., Zheng, A. & Walensky, R. P. Assessment of SARS-CoV-2 Screening Strategies to Permit the Safe Reopening of College Campuses in the United States. *JAMA Netw. Open* **3**, e2016818 (2020).

6. Isolation. *Centers for Disease Control and Prevention* https://www.cdc.gov/coronavirus/2019-ncov/your-health/isolation.html (2023).

7. He, X. *et al.* Temporal dynamics in viral shedding and transmissibility of COVID-19. *Nat. Med.* **26**, 672–675 (2020).

8. Bjørnstad, O. N. *Epidemics: Models and Data Using R*. (Springer International Publishing, Cham, 2018). doi:10.1007/978-3-319-97487-3.

9. Kucirka, L. M., Lauer, S. A., Laeyendecker, O., Boon, D. & Lessler, J. Variation in False-Negative Rate of Reverse Transcriptase Polymerase Chain Reaction–Based SARS-CoV-2 Tests by Time Since Exposure. *Ann. Intern. Med.* **173**, 262–267 (2020).

10. Surkova, E., Nikolayevskyy, V. & Drobniewski, F. False-positive COVID-19 results: hidden problems and costs. *Lancet Respir. Med.* **8**, 1167–1168 (2020).

11. Altarawneh, H. N. *et al.* Effects of Previous Infection and Vaccination on Symptomatic Omicron Infections. *N. Engl. J. Med.* **387**, 21–34 (2022).

12. Andrews, N. *et al.* Covid-19 Vaccine Effectiveness against the Omicron (B.1.1.529) Variant. *N. Engl. J. Med.* **386**, 1532–1546 (2022).
